# Synthesis and Broad-Spectrum Antiviral Evaluation of Novel 1,2,3-Triazole–Quinoline Derivatives Targeting SARS-CoV-2, Influenza, DENV-2, and CHIKV

**DOI:** 10.3390/v18090951

**Published:** 2026-08-31

**Authors:** Alcione Silva de Carvalho, Natalia Fintelman-Rodrigues, Victória Toledo Diniz de Camargo, Lina Silva-Trujillo, Caroline Souza de Freitas, Isabela Alencar Graciano, Acácio Silva de Souza, Camilla Blanco de Assis, Otávio Augusto Chaves, Fernando de Carvalho da Silva, Vitor F. Ferreira, Thiago Moreno L. Souza

**Affiliations:** 1 Programa de Pós-Graduação em Ciências Aplicadas a Produtos para a Saúde, Faculdade de Farmácia, Universidade Federal Fluminense, Niterói 24241-000, RJ, Brazil; 2 Laboratory of Immunopharmacology, Center for Research, Innovation, and Surveillance in COVID-19 and Health Emergencies, Oswaldo Cruz Institute, Rio de Janeiro 21040-361, RJ, Brazil; 3 Center for Technological Development in Health, National Institute for Science and Technology on Innovation on Neglected Diseases and Neglected Populations, Oswaldo Cruz Foundation, Rio de Janeiro 21040-900, RJ, Brazil; 4 Laboratory of Viral Diseases Immunology, René Rachou Institute, Oswaldo Cruz Foundation, Belo Horizonte 30190-002, MG, Brazil; 5 Applied Molecular Chemistry Laboratory (LaQMA), Department of Chemistry and Environmental Sciences, Institute of Biosciences, Humanities and Exact Sciences (IBILCE), São Paulo State University (UNESP), São José do Rio Preto 15054-000, SP, Brazil; 6 Departamento de Química Orgânica, Instituto de Química, Universidade Federal Fluminense, Niterói 24020-141, RJ, Brazil; 7 Graduation Program on Surveillance in Health, Iguaçú University, Nova Iguaçu 26260-045, RJ, Brazil

**Keywords:** molecular hybridization, antiviral discovery, broad-spectrum antiviral agents

## Abstract

The persistent global threat posed by emerging and re-emerging RNA viruses, including SARS-CoV-2, influenza A(H1N1)pdm09, DENV-2, and CHIKV, highlights the critical need for novel and broad-spectrum antiviral agents. Building upon the established antiviral activity of the quinoline scaffold, this study employed a molecular hybridization strategy to design and synthesize a novel series of 1,2,3-triazole–quinoline derivatives (compounds **4a**–**4h**). This approach strategically fused the bioactive quinoline nucleus with the pharmaceutically favorable 1,2,3-triazole ring. The new compounds were synthesized efficiently in three steps with moderate-to-high yields, and their structures were subsequently determined. Their inhibitory capacities against the four viruses and cytotoxicity across relevant cell lines were evaluated in vitro. The initial screening demonstrated that compounds **4a**–**4h** possessed broad-spectrum antiviral activity, showing high inhibition percentages at 10 µM against SARS-CoV-2, DENV-2, and CHIKV. Notably, compounds **4c** and **4e**, substituted with methoxy and ethyl groups, respectively, displayed exceptional potency and safety against SARS-CoV-2, yielding high selectivity indices (SI: 1896.5 and 1265.8, respectively). Furthermore, in silico absorption, distribution, metabolism, and excretion (ADME) and molecular docking calculations suggested favorable drug-likeness profiles, including improved lipophilicity and non-P-glycoprotein substrate feasibility. The findings validate the molecular hybridization strategy, establishing this new class of compounds as promising, multi-target antiviral prototypes for further preclinical exploration against prevalent viral pathogens.

## 1. Introduction

Infectious diseases have threatened global populations for centuries. These threats are primarily represented by ribonucleic acid (RNA) viruses, which possess a substantial ability to mutate and recombine/reassort [1,2], leading to variants of public health concern. Following major epidemics or pandemics, viruses such as the 2009 pandemic influenza A(H1N1) virus (influenza A(H1N1)pdm09), severe acute respiratory syndrome coronavirus 2 (SARS-CoV-2), chikungunya virus (CHIKV), and dengue virus (DENV) have continued to circulate worldwide, causing a substantial burden of respiratory and arboviral diseases [3,4].

Although vaccines are available for some of these threats, novel variants of the influenza A virus and SARS-CoV-2 continuously emerge and escape vaccine-induced immune responses. DENV and CHIKV can also be prevented with newer vaccines, but phase 4 trials are currently ongoing to ensure homogeneous efficacy across different serotypes and genotypes [5,6,7]. Considering that respiratory viruses like influenza A and SARS-CoV-2 can cause severe acute respiratory illness, and arboviruses like CHIKV and DENV can range from causing fever and arthralgia to severe neurological and hemorrhagic manifestations [5], effective antivirals are urgently needed as complementary tools to combat these pathogens.

The drug repositioning (or repurposing) strategy is widely used in the search for active therapeutic agents against various diseases. Among these repurposed options, remdesivir (RDV) gained prominence against SARS-CoV-2. RDV was shown to be clinically effective when administered soon after the onset of coronavirus disease 2019 (COVID-19); however, access to this drug remains limited even in wealthy countries, and its requirement for intravenous administration makes it impractical for early outpatient use [8]. Subsequently, Paxlovid [9,10,11], an orally available main protease (M^pro^) inhibitor of SARS-CoV-2, proved to be an effective compound and has been widely deployed against COVID-19.

**Figure 1:**
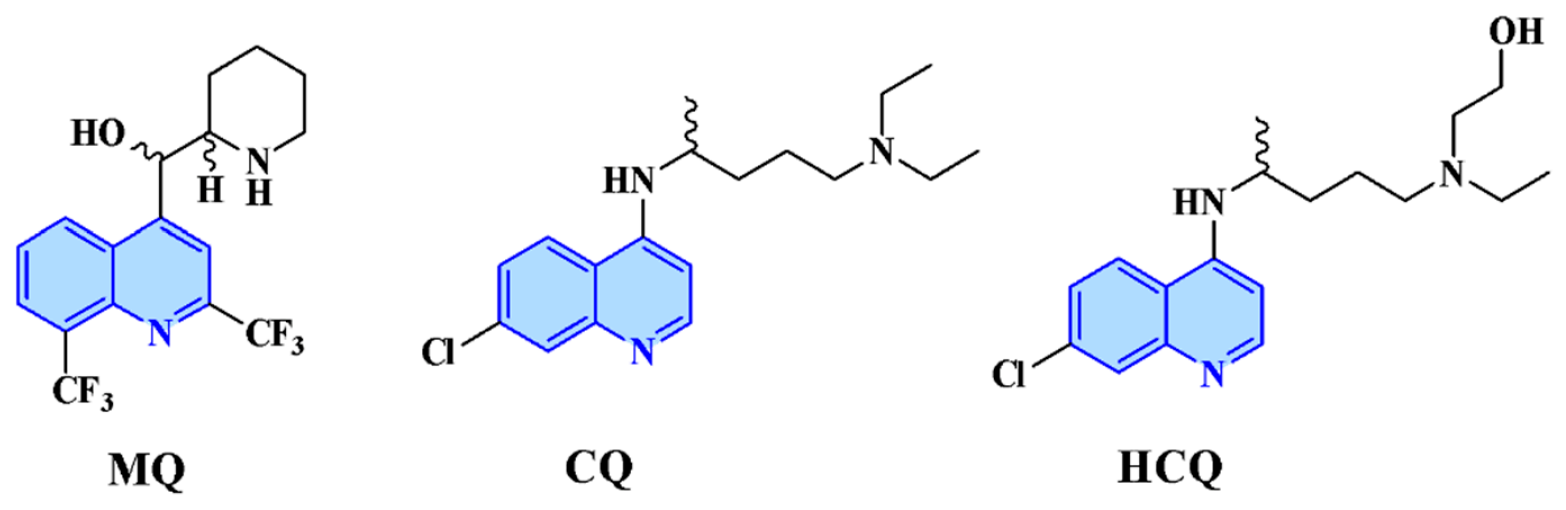
Structures of some compounds belonging to the quinoline family, i.e., mefloquine (**MQ**), chloroquine (**CQ**), and hydroxychloroquine (**HCQ**).

Regarding repurposed drugs from the quinoline family (Figure 1), we previously demonstrated the in vitro anti-SARS-CoV-2 activity of mefloquine (MQ) in human respiratory cells through the inhibition of viral endocytosis [12]. In this same cellular model, chloroquine (CQ) and hydroxychloroquine (HCQ) were inactive [12], consistent with broad clinical observations during the COVID-19 pandemic. Quinoline derivatives have already been tested against arboviruses such as *Orthoflavivirus* (DENV and Zika virus) and *Alphavirus* (CHIKV), exhibiting inhibition values in the micromolar range in cell-based assays, reinforcing the promising action of compounds in this class [13,14,15].

**Figure 2:**
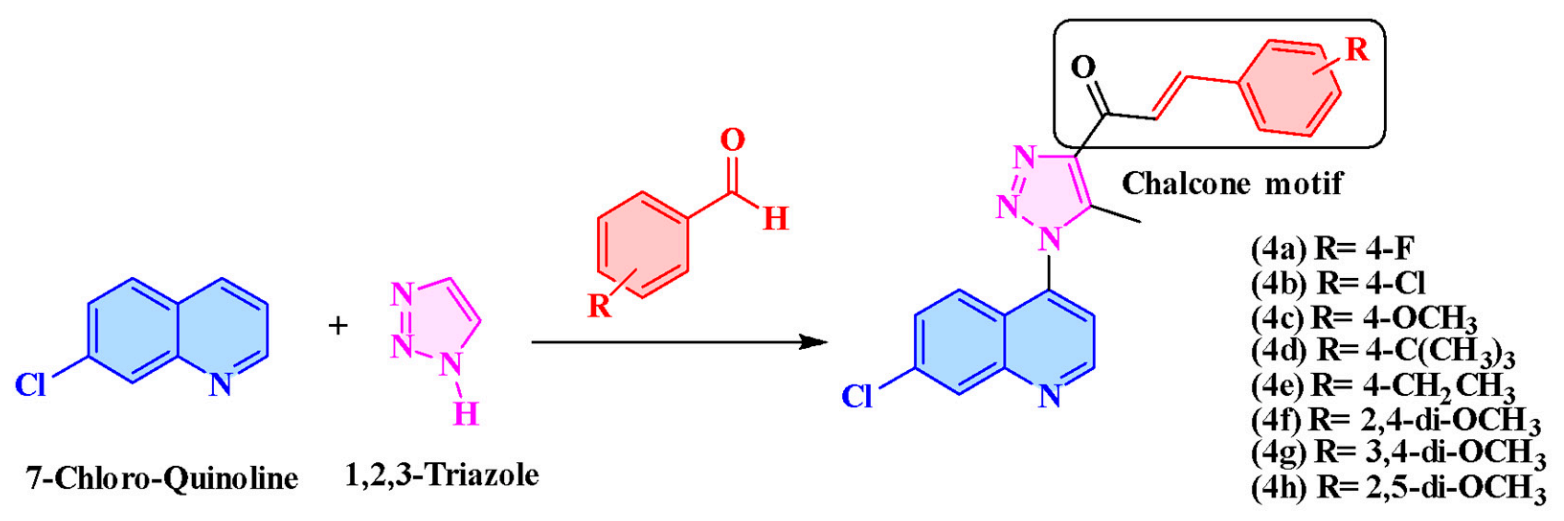
Design of 1,2,3-triazole–quinoline derivatives containing a chalcone motif (**4a**–**4h**) using a hybridization strategy.

To innovate within the quinoline scaffold and develop novel antiviral agents targeting key processes in viral replication, the present work couples the quinoline nucleus with a 1,2,3-triazole ring (Figure 2), a prominent pharmacophore among nitrogen-containing heterocycles. This five-membered ring, bearing three nitrogen atoms, is synthetically accessible and functions as a versatile linker—a structural property widely exploited in designing lead compounds for diverse biological targets, including antivirals [16,17,18]. The 1,2,3-triazole core offers several distinct advantages in drug design, including hydrogen-bond acceptor sites that enable key interactions with biomolecular targets through hydrogen bonding, π-π stacking, and dipole–dipole interactions. Moreover, its high chemical and metabolic stability under biological conditions contributes to favorable pharmacokinetic profiles and reduced toxicity [19,20,21]. Building upon the relevance of the quinoline scaffold as an anti-SARS-CoV-2 archetype and the proven utility of 1,2,3-triazoles as antiviral moieties, this study extends our efforts to identify novel active compounds. Herein, we report the rational design, synthesis, and biological evaluation of novel antiviral candidates based on a molecular hybridization strategy [22]. This approach combines two distinct bioactive fragments within a single molecular entity to generate novel functional prototypes. Accordingly, quinoline and 1,2,3-triazole pharmacophores were integrated to yield a series of 1,2,3-triazole–7-chloroquinoline derivatives containing a chalcone subunit with varied phenyl substituents (**4a**–**4h**, Figure 2). These compounds were evaluated in vitro for cytotoxicity and antiviral activity against SARS-CoV-2, influenza A(H1N1)pdm09, dengue virus serotype 2 (DENV-2), and CHIKV.

## 2. Materials and Methods

### 2.1. Chemistry

Reagents and solvents were purchased from commercial suppliers, including Sigma-Aldrich/Merck (Sigma-Aldrich, St. Louis, MO, USA), and were used without further purification unless otherwise stated. Reaction progress was monitored by thin-layer chromatography (TLC) using Merck silica gel 60 F254 plates supported on 0.20 mm aluminum sheets, with visualization under UV light (Sigma-Aldrich, St. Louis, MO, USA). Melting points were determined on a Büchi Model B-545 apparatus (Büchi Labortechnik AG, Flawill, Switzerland) and were reported without correction. The HRMS analyses were performed using an LC-MS Bruker Daltonics MicroTOF time-of-flight analyzer (Bruker Daltonics, Billerica, MA, USA). Fourier transform infrared (FTIR) spectra were recorded on a Thermo Scientific Nicolet 6700 FTIR spectrophotometer using the attenuated total reflection (ATR) technique (Thermo Fisher Scientific, Waltham, MA, USA). ^1^H and ^13^C NMR spectra were acquired at 500.00 and 126.00 MHz, respectively, using a Bruker Avance spectrometer equipped with a 5 mm probe (Bruker Corporation, Billerica, MA, USA). Chemical shifts (δ) were reported in parts per million (ppm) relative to the residual solvent signal, and coupling constants (J) were expressed in hertz (Hz).

#### 2.1.1. General Procedure for the Synthesis of 4-Azido-7-Chloroquinoline (**2**)

In a 50 mL flask, 20 mmol of sodium azide (2 eq.), 10 mmol of 4,7-dichloroquinoline (1 eq.), and 10 mL of dry dimethylformamide (DMF) were stirred at 60 °C for 6 h. The reaction was monitored by TLC using ethyl acetate:hexane (40:60, *v*/*v*) as eluent. After completion of the reaction, the mixture was allowed to cool to room temperature, and water was added. Then, the resulting mixture was extracted with dichloromethane. The combined organic phase was washed with saturated salt water, dried over anhydrous sodium sulfate (Na_2_SO_4_), filtered, and concentrated under reduced pressure to give the corresponding product, which was purified by flash column chromatography with acetate:hexane (2:8). A white solid (68% yield) was obtained with a m.p. of 110–112 °C.

Compound **2**: IR (cm^–1^): 3078, 2117, 1578, 1199, 817 cm^−1^. ^1^H NMR (500.00 MHz, CDCl_3_, δ in ppm): 7,14 (1H, d, J = 4.8 Hz; 3-H), 7.48 (1H, dd, J = 8.8, 2.0 Hz; 6-H), 7.98 (1H, d, J = 8.8 Hz; 5-H), 8.11 (1H, d, J = 2.0 Hz; 8-H), 8.86 (1H, d, J = 4.8 Hz; 2-H). ^13^C NMR (CDCl_3_, δ in ppm): 108.7, 119.9, 123.7, 127.5, 128.1, 136.5, 146.3, 149.5, 151.2.

#### 2.1.2. General Procedure for the Synthesis of 1-(1-(7-Chloroquinolin-4-yl)-5-Methyl-1H-1,2,3-Triazol-4-yl)Ethan-1-One (**3**)

In a 50 mL flask, 0.9 mmol (1 eq.) of 4-azido-7-chloroquinoline was added to 1.6 mmol (1.8 eq.) of 4-acetylacetone, 5 mmol (5.6 eq.) of potassium carbonate (K_2_CO_3_), and 6 mL of ethanol (EtOH). The mixture was stirred at 70 °C for 1 h. After completion of the reaction, monitored by TLC using ethyl acetate:hexane (70:30, *v*/*v*) as eluent, the reaction mixture was allowed to cool to room temperature and was completely evaporated under reduced pressure. Then, the mixture was diluted with ice-cold water, and its pH was adjusted to 6 with 6 M HCl solution. The precipitate formed was collected by filtration using a pump, washed with water, and dried. The product was crystallized from EtOH to yield a pure white solid (74% yield) with a m.p. of 97–99 °C.

Compound **3**: IR (cm^−1^): 3048; 1681; 1431; 639. ^1^H NMR (500 MHz, CDCl_3_, δ in ppm): 3.36 (s, 3H, CH_3_), 3.39 (s, 3H, COCH_3_), 7.50 (d, J = 9,0 Hz, 1H, H5); 7.74 (dd, J = 9,0 Hz, 1H, H6), 7.86 (d, J = 4,5 Hz, 1H, H3); 8.30 (d, J = 2,1 Hz, 1H, H8); 9.11 (d, J = 4,6 Hz, 1H, H2). ^13^C (100 MHz, CDCl_3_, δ in ppm): 9.69, 27.56, 108.7, 119.9, 121.40, 123.8, 127.9, 131.45, 136.9, 141.20, 146.8, 149.1, 150.9, 190.26.

#### 2.1.3. General Procedure for the Synthesis of Compound (E)-1-(1-(7-Chloroquinolin-4-yl)-5-Methyl-1H-1,2,3-Triazol-4-yl)-3-(R)prop-2-en-1-One (**4a**–**4h**)

In a round-bottom flask, a mixture of 10 mmol of 1-(1-(7-chloroquinolin-4-yl)-5-methyl-1*H*-1,2,3-triazol-4-yl)ethan-1-one (**3**) and 12 mmol of the appropriate substituted aromatic aldehyde in 70 mL of EtOH was added slowly to a solution of 12.8 mmol of potassium hydroxide in 10 mL of water. The mixture was stirred in an ice bath for 2 h and then at room temperature for 24 h. The progress of the reaction was monitored by TLC using ethyl acetate:hexane (60:40, *v*/*v*) as eluent. After completion of the reaction, the mixture was filtered, and the obtained solid was washed with cold water. The product was crystallized from EtOH to yield pure derivatives **4a**–**4h** in good yield. The spectral data used to structurally characterize **4a**–**4h** are summarized in the Supplementary Materials, while the main signals and physical characteristics are shown below:

(*E*)-1-(1-(7-Chloroquinolin-4-yl)-5-Methyl-1*H*-1,2,3-Triazol-4-yl)-3-(4-Fluorophenyl) prop-2-en-1-one (**4a**): White solid in 85% yield, m.p. 195–197 °C. IR (cm^−1^): 1664, 1593, 826–673. ^1^H NMR (500 MHz, DMSO- d_6_, δ in ppm): 9.21 (d, J = 4.6 Hz, 1H, H2), 8.30 (d, J = 2.1 Hz, 2H, H8), 7.96 (d, J = 16.1 Hz, 1H, H5′ or H6′), 7.90 (d, J = 16.2 Hz, 1H, H5′ or H6′); 7.90–7.88 (m, 2H), 7.86 (d, J = 4.5 Hz, 1H, H3), 7.73 (dd, J = 9.0, 2.2 Hz, 1H, H6), 7.50 (d, J = 9.0 Hz, 1H, H5), 7.30 (t, J = 8.8 Hz, 2H), 2.07 (s, 3H, CH_3_). ^13^C NMR(126 MHz, DMSO- d_6_, δ in ppm): 183.77; 165.72, 162.41, 152.85, 149.79, 143.56, 142.79, 141.29, 139.40, 136.25, 131.60, 131.55, 131.44, 129.87, 129.82, 128.64, 125,2; 125.13, 123.47, 123.44, 122.42, 120.32, 116.73, 116.73, 9.91. HRMS (ESI): calc. for C_21_H_14_ClFN_4_O 415.0732; found [M + Na]^+^ 415.0720.

(*E*)-3-(4-Chlorophenyl)-1-(1-(7-Chloroquinolin-4-yl)-5-Methyl-1*H*-1,2,3-Triazol-4-yl)prop-2-en-1-one (**4b**): White solid in 58% yield, m.p. 207–208 °C. IR (cm^−1^): 2957, 1583, 1511, 847–673; ^1^H NMR (500 MHz, DMSO- d_6_, δ in ppm): 9.22 (d, J = 4.5 Hz, 1H, H2), 8.31 (d, J = 2.2 Hz, 1H, H8), 8.02 (d, J = 16.0 Hz, 1H, H5′), 7.89 (d, J = 16.0 Hz, 1H, H6′), 7.87–7.83 (m, 3H, H3 and H2″ and H6″), 7.74 (dd, J = 9.0, 2.2 Hz, 1H, H6), 7.54 (d, J = 8.5 Hz, 2H, H3” and H5″), 7.50 (d, J = 9.0 Hz, 1H, H5), 2.08 (s, 3H, CH_3_). ^13^C NMR(126 MHz, DMSO- d_6_, δ in ppm): 183.20, 152.32, 149.25, 143.00, 140.84, 138.85, 135.71, 135.35, 133.32, 130.27, 129.34, 129.09, 128.10, 124.68, 123.70, 121.87, 119.79, 9.38. HRMS (ESI): calc. for C_21_H_14_Cl_2_N_4_O 431.0436; found [M + Na]^+^ 431.0434.

(*E*)-1-(1-(7-Chloroquinolin-4-yl)-5-Methyl-1*H*-1,2,3-Triazol-4-yl)-3-(4-Methoxyphenyl)prop-2-en-1-one (**4c**): Yellow solid in 88% yield, m.p. 134–135 °C. IR (cm^−1^): 2971, 1659, 1584, 829–636. ^1^H NMR (500 MHz, DMSO- d_6_, δ in ppm): 9.21 (d, J = 4.6 Hz, 1H, H2), 8.30 (d, J = 2.1 Hz, 1H, H8), 7.99 (d, 1H, J = 16.0 Hz, 1H, H5′), 7.93 (d, 1H, J = 16.0 Hz, 1H, H6′), 7.88 (s, 1H), 7.86 (d, J = 4.5 Hz, 1H, H3), 7.78 (d, J = 8.8 Hz, 2H), 7.73 (dd, J = 9.0, 2.1 Hz, 1H, H6), 7.49 (d, J = 9.0 Hz, 1H, H5), 7.04 (d, J = 8.8 Hz, 2H), 3.83 (s, 3H, OCH_3_), 2.49 (s, 3H, CH_3_). ^13^C NMR(126 MHz, DMSO- d_6_, δ in ppm): 183.78, 162.17, 152.85, 149.78, 143.97, 143.69, 141.00, 139.45, 136.24, 131.04, 129.86, 128.62, 127.57, 125.21, 122.44, 121.12, 120.31, 115.21, 114.68, 55.93, 10.01. HRMS (ESI): calc. for C_22_H_17_ClN_4_O_2_ 427.0932; found [M + Na]^+^ 427.0929.

(*E*)-3-(4-(Tert-Butyl)phenyl)-1-(1-(7-Chloroquinolin-4-yl)-5-Methyl-1*H*-1,2,3-Triazol-4-yl)prop-2-en-1-one (**4d**): Yellow solid in 94% yield, m.p. 105–107 °C. IR (cm^−1^): 2971, 1659, 1584, 829–636. ^1^H NMR (500 MHz, DMSO- d_6_, δ in ppm): 9.21 (d, J = 4.6 Hz, 1H, H2), 8.30 (d, J = 2.1 Hz, 1H, H8), 7.97 (d, J = 16.0 Hz, 1H, H5′ or H6′), 7.89 (d, J = 16.0 Hz, 1H, H5′ or H6′), 7.86 (d, J = 4.6 Hz, 1H, H3), 7.74 (d, J = 8.2 Hz, 2H), 7.72 (d, J = 2.1 Hz, 1H), 7.50 (dd, J = 8.8, 3,1 Hz, 3H), 2.19 (s, 3H, CH_3_), 1.31 (s, 9H, C(CH_3_)_3_). ^13^C NMR(126 MHz, DMSO- d_6_, δ in ppm): 180.78, 160.17, 151.52, 148.78, 145.97, 141.69, 141.00, 139.45, 136.24, 131.04, 129.86, 128.62, 127.57, 124.12, 121.12, 123.17, 35.46, 31.51, 29.20, 10.56. HRMS (ESI): calc. for C_25_H_23_ClN_4_O 453.1452; found [M + Na]^+^ 453.1434.

(*E*)-1-(1-(7-Chloroquinolin-4-yl)-5-Methyl-1*H*-1,2,3-Triazol-4-yl)-3-(4-Ethylphenyl)prop-2-en-1-one (**4e**): White solid in 92% yield, m.p. 149–151 °C. IR (cm^−1^): 2971, 1659, 1584, 829–636. ^1^H NMR (500 MHz, DMSO- d_6_, δ in ppm): 9.21 (d, J = 4.6 Hz, 1H, H2), 8.30 (d, J = 2.1 Hz, 1H, H8), 7.97 (d, J = 16.0 Hz, 1H, H5′ or H6′), 7.88 (d, J = 16.0 Hz, 1H, H5′ or H6′), 7.86 (d, J = 4.6 Hz, 1H, H3), 7.73 (dd, J = 7.9, 2.1 Hz, 2H), 7.50 (d, J = 9.0 Hz, 1H), 7.33 (d, J = 7.9 Hz, 2H), 2.66 (q, J = 7.6 Hz, 2H, CH_2_-CH_3_), 2.08 (s, 3H, CH_3_), 1.21 (t, J = 7.6 Hz, 3H, CH_2_-CH_3_). ^13^C NMR(126 MHz, DMSO- d_6_, δ in ppm): 183.85, 152.84, 149.77, 147.76, 144.10, 143.62, 141.17, 139.41, 136.24, 132.43, 129.86, 129.25, 129.02, 128.61, 125.21, 122.55, 122.41, 120.31, 40.01, 15.51, 9.90. HRMS (ESI): calc. for C_23_H_19_ClN_4_O 425.1139, found [M + Na]^+^ 425.1121.

(*E*)-1-(1-(7-Chloroquinolin-4-yl)-5-Methyl-1*H*-1,2,3-Triazol-4-yl)-3-(2,4-Dimethoxyphenyl)prop-2-en-1-one (**4f**): Yellow solid in 68% yield, m.p. 170–172 °C. IR (cm^−1^): 3046, 1655, 1294, 1162, 838–672. ^1^H NMR (500 MHz, DMSO- d_6_, δ in ppm): 9.21 (d, J = 4.6 Hz, 1H, H2), 8.29 (d, J = 2.2 Hz, 1H, H8), 8.09 (d, J = 16,1 Hz, 1H, H5′), 7.94 (d, J = 16.0 Hz, 1H, H6′), 7.85 (d, J = 4.5 Hz, 1H, H3), 7.75 (d, J = 8.3 Hz, 1H), 7.72 (dd, J = 9.0, 2.1 Hz, 1H, H6), 7.49 (d, J = 9.0 Hz, 1H, H5), 6.67 (s, 1H), 6.65 (d, J = 2.4 Hz, 1H), 3.93 (s, 3H, OCH_3_), 3.85 (s, 3H, OCH_3_), 2.48 (s, 3H, CH_3_), 2.07 (s, 1H). ^13^C NMR(126 MHz, DMSO- d_6_, δ in ppm): δ 183.27, 163.01, 160.04, 152.03, 148.97, 143.00, 140.02, 138.68, 138.40, 135.42, 130.29, 129.04, 127.80, 124.42, 121.64, 120.27, 119.49, 115.68, 106.41, 98.35, 55.63, 55.26, 9.17. HRMS (ESI): calc. for C_23_H_19_ClN_4_O_3_: 457.1037, found [M + Na]^+^ 457.1036.

(*E*)-1-(1-(7-Chloroquinolin-4-yl)-5-Methyl-1*H*-1,2,3-Triazol-4-yl)-3-(3,4-Dimethoxyphenyl)prop-2-en-1-one (**4g**): Yellow solid in 72% yield, m.p. 188–189 °C. IR (cm^−1^): 2998, 1588,1509, 1264, 805–767. ^1^H NMR (500 MHz, DMSO- d_6_, δ in ppm): 9.21 (d, J = 4.6 Hz, 1H, H2), 8.30 (d, J = 2.1 Hz, 1H, H8), 7.90 (d, J = 16.1 Hz, 1H, H5′ or H6′), 7.86 (d, J = 4.5 Hz, 1H, H3), 7.86 (d, J = 16.1 Hz, 1H, H5′ or H6′), 7.73 (dd, J = 9.0, 2.1 Hz, 1H, H6), 7.49 (d, J = 9.0 Hz, 1H, H5), 7.41 (d, J = 2.1 Hz, 1H, H2″), 7.39 (dd, J = 8.3, 2.1 Hz, 1H, H6″), 7.06 (d, J = 8.3 Hz, 1H, H5″), 3.87 (s, 3H, H7′ or H8′), 3,84 (s, 3H, H7′ or H8′), 2,49 (s, 3H, CH_3_), 2,08 (s, 1H). ^13^C NMR(126 MHz, DMSO- d_6_, δ in ppm): 183.80, 152.85, 152.19, 149.78, 144.43, 143.69, 141.00, 139.45, 136.23, 129.85, 128.62, 127.85, 125.21, 123.91, 122.44, 121.30, 120.30, 112.68, 111.83, 56.34, 56.28, 56.19, 9.90. HRMS (ESI): calc. for C_23_H_19_ClN_4_O_3_: 457.1037, found [M + Na]^+^ 457.1035.

(*E*)-1-(1-(7-Chloroquinolin-4-yl)-5-Methyl-1*H*-1,2,3-Triazol-4-yl)-3-(2,5-Dimethoxyphenyl)prop-2-en-1-one (**4h**): Yellow solid in 62% yield, m.p. 124–126 °C. IR (cm^−1^): 3048, 1611,1504, 1234, 814–771. ^1^H NMR (500 MHz, DMSO- d_6_, δ in ppm): 9.21 (d, J = 4.6 Hz, 1H, H2), 8.30 (d, J = 2.1 Hz, 1H, H8), 8.12 (d, J = 16.2 Hz, 1H, H5′ or H6′), 8.04 (d, J = 16.2 Hz, 1H, H5′ or H6′), 7.86 (d, J = 4.5 Hz, 1H, H3), 7.73 (dd, J = 9.0, 2.1 Hz, 1H, H6), 7.50 (d, J = 9.0 Hz, 1H, H5), 7.33 (d, J = 2.8 Hz, 1H, H6″), 7.09 (d, J = 9.0 Hz, 1H, H3”), 7.06 (dd, J = 9.0, 2,8 Hz, 1H, H4”), 3.88 (s, 3H, OCH_3_), 3,80 (s, 3H, OCH_3_), 2,08 (s, 3H, CH_3_). ^13^C NMR(126 MHz, DMSO- d_6_, δ in ppm): 183.60, 153.40, 153.11, 152.33, 149.25, 143.14, 140.67, 138.90, 138.34, 135.71, 129.33, 128.10, 124.70, 123.53, 123.50, 121.90, 119.79, 118.42, 113.50, 113.14, 56.33, 55.72, 55.51, 9.39. HRMS (ESI): calc. for C_23_H_19_ClN_4_O_3_: 457.1037, found [M + Na]^+^ 457.1036.

### 2.2. Virology

#### 2.2.1. Cells

The African green monkey kidney cells (Vero, E6 subtype), human pulmonary epithelial cells (Calu-3) (both donated by Rio de Janeiro’s cell bank; BCRJ, Rio de Janeiro, Brazil), and Madin-Darby canine kidney (MDCK) cells (donated by Influenza Reagent Resource; International Reagent Resource, IRR, Centers for Disease Control and Prevention, USA) were cultured in high-glucose Dulbecco’s Modified Eagle Medium (DMEM) (Thermo Fisher, Cat. #10313021). On the other hand, human hepatoma cells (Huh-7) (donated by BCRJ cell bank, Brazil) were cultured in low-glucose DMEM (Thermo Fisher, Cat. #318549). All culture media were supplemented with 10% fetal bovine serum (FBS) (Hyclone, Logan, UT, USA, Cat. #SH30396.03HI), 100 U/mL of penicillin, and 100 μg/mL of streptomycin (P/S) (Thermo Fisher, Cat. #15070063). The cells were cultured at 37 °C in a humid atmosphere with 5% CO_2_.

#### 2.2.2. Cytotoxicity Assays

Cytotoxicity assays for arboviruses, DENV-2 (strain 16881) and CHIKV (donated by Amilcar Tanuri from Federal University of Rio de Janeiro, Brazil), were both performed on Huh-7 cells (2 × 10^4^ cells/well). For influenza A(H1N1)pdm09 (donated by IRR, USA) and SARS-CoV-2 (GenBank #MT710714; Institutional Review Board approval, 30650420.4.1001.0008), the assays were performed on MDCK cells (1 × 10^4^ cells/well) and Calu-3 cells (2 × 10^5^ cells/well), respectively. Huh-7 cells were treated with different concentrations of **3**, **4a**–**4h** (5, 10, 50, and 100 µM), whereas MDCK and Calu-3 cells were treated with different concentrations of **3**, **4a–4h** (10, 50, 100, 300, and 500 µM), prepared in low- or high-glucose DMEM, depending on the cellular lineage. Cells were maintained in maintenance medium (1 g/L) supplemented with 2% FBS and 1% P/S. The cells were incubated for 48 h in a 5% CO_2_ atmosphere at 37 °C. Subsequently, 100 µL of the culture supernatant was removed, and 20 µL of resazurin (Promega, Madison, WI, USA, Cat. # G8080) was added. After 2–4 h of incubation, the reading was performed on a GloMax fluorometer plate reader (Promega, Madison, WI, USA), with 560 nm excitation and 590 nm emission, following the literature [23]. Nonlinear regression analysis of the dose–response curves was performed to determine the half-maximal cytotoxic concentration (CC_50_) in GraphPad Prism software (version 10.0, San Diego, CA, USA).

#### 2.2.3. Viral Replication Inhibition Assays

Viral replication inhibition assays were performed in 96-well culture plates (2 × 10^5^ Calu-3 cells/well for SARS-CoV-2; 2 × 10^4^ MDCK cells/well for influenza A(H1N1)pdm09; and 2 × 10^4^ Huh-7 cells/well for both DENV-2 and CHIKV). Cells were infected with a multiplicity of infection (MOI) of 0.1 for 1 h at 37 °C in a 5% CO_2_ atmosphere. After incubation, the inoculum was removed, and the cells were treated with different compounds (10 µM) and maintained for 24 h for influenza A(H1N1)pdm09 or 48 h for SARS-CoV-2, DENV-2, and CHIKV at 37 °C in a 5% CO_2_ atmosphere. Subsequently, the supernatants containing the virus were collected for quantification of viral replication by real-time RT-PCR for influenza A(H1N1)pdm09, DENV-2, and CHIKV, while plaque assays were performed for SARS-CoV-2. After quantification, the percentage of inhibition of the compounds was calculated in comparison with the infected and untreated controls. Compounds showing antiviral activity in the primary screening at 10 μM were subsequently evaluated over a range of concentrations to generate dose–response curves. Viral replication was quantified as described above, and the percentage of inhibition at each concentration was determined relative to the infected untreated control. Half-maximal effective concentration (EC_50_) values were calculated by nonlinear regression analysis using a four-parameter dose–response model in GraphPad Prism software (version 10.0, San Diego, CA, USA). For the compounds in which replicates showed a rate equal to or above 20% variability, the half-maximal effective concentration (EC_50_) value was considered not determined (ND).

#### 2.2.4. Quantification of Viral Titers by Plaque Assays

The viral titer quantification of the SARS-CoV-2 assay supernatants in Calu-3 cells was determined by plaque assay titration. Initially, Vero-E6 cells were seeded in 96-well plates (2 × 10^4^ cells/well) and incubated with serial dilutions of the biological supernatant. After 1 h of incubation in a 5% CO_2_ atmosphere at 37 °C, 50 µL of culture medium supplemented with 2.4% carboxymethylcellulose was added, and the cells were incubated again for 72 h. To perform the counting of plaque-forming units, the cells were fixed for 2 h at room temperature with 100 µL of 10% formalin solution. For visualization of plaque formation, the plates were washed with 100 µL of 0.04% crystal violet for 1 h at room temperature. Cell lysis plaques were counted, and the viral titers were determined in plaque-forming units per mL (PFU/mL).

#### 2.2.5. Quantification of Viral RNA by Real-Time RT-PCR

Total RNA present in the supernatants of the in vitro infection assays for influenza A(H1N1)pdm09, DENV-2, and CHIKV was extracted using the Maxwell^®^ RSC Viral Total Nucleic Acid Purification Kit, according to the manufacturer’s instructions. Quantitative RT-PCR was conducted using the GoTaq^®^ Probe 1-Step RT-qPCR System (Promega, Madison, WI, USA) and thermal cycling conditions following the manufacturer’s instructions, on a QIAquant 96 detection platform (Qiagen, Germantown, MD, USA). Each 10 µL reaction mixture comprised 7.5 µL of reaction mix buffer, 0.3 µL of enzyme mix, 20 µM of each primer, 10 µM of the probe, and 5 µL of RNA template. The primers and probes were previously described [24]. Viral quantification was performed using the standard curve method.

### 2.3. Molecular Modeling

#### 2.3.1. Absorption, Distribution, Metabolism, and Excretion (ADME) Prediction

The ADME, physicochemical, drug-like, and related parameters were estimated for **3** and **4a**–**4h** with the free web server SwissADME (http://www.swissadme.ch/index.php, accessed on 15 May 2026) [25].

#### 2.3.2. Molecular Docking Calculations

The three-dimensional structures of two human isoforms of cytochrome P450 (CYP450) were obtained in the Protein Data Bank (PDB) with the access codes 1R9O and 4GQS. The chemical structures of **3** and **4a**–**4h** were built and energy-minimized with Spartan’18 software (Wavefunction Inc., Irvine, CA, USA) [26] using the Density Functional Theory (DFT) method. The molecular docking calculations were performed with GOLD 2024.3.0 software (Cambridge Crystallographic Data Centre, Cambridge, CB2 1EZ, UK) [27]. Molecular docking calculations were carried out for an 8 Å radius around each selected binding pocket, and the standard scoring function ChemPLP was used due to the best reported correlation between in silico and experimental data for CYP450 [28]. The 3D figures were generated with PyMOL Molecular Graphics System 1.0 level software (Delano Scientific LLC software, Schrödinger, New York, NY, USA) [29].

## 3. Results

### 3.1. Chemistry

**Scheme 1:**
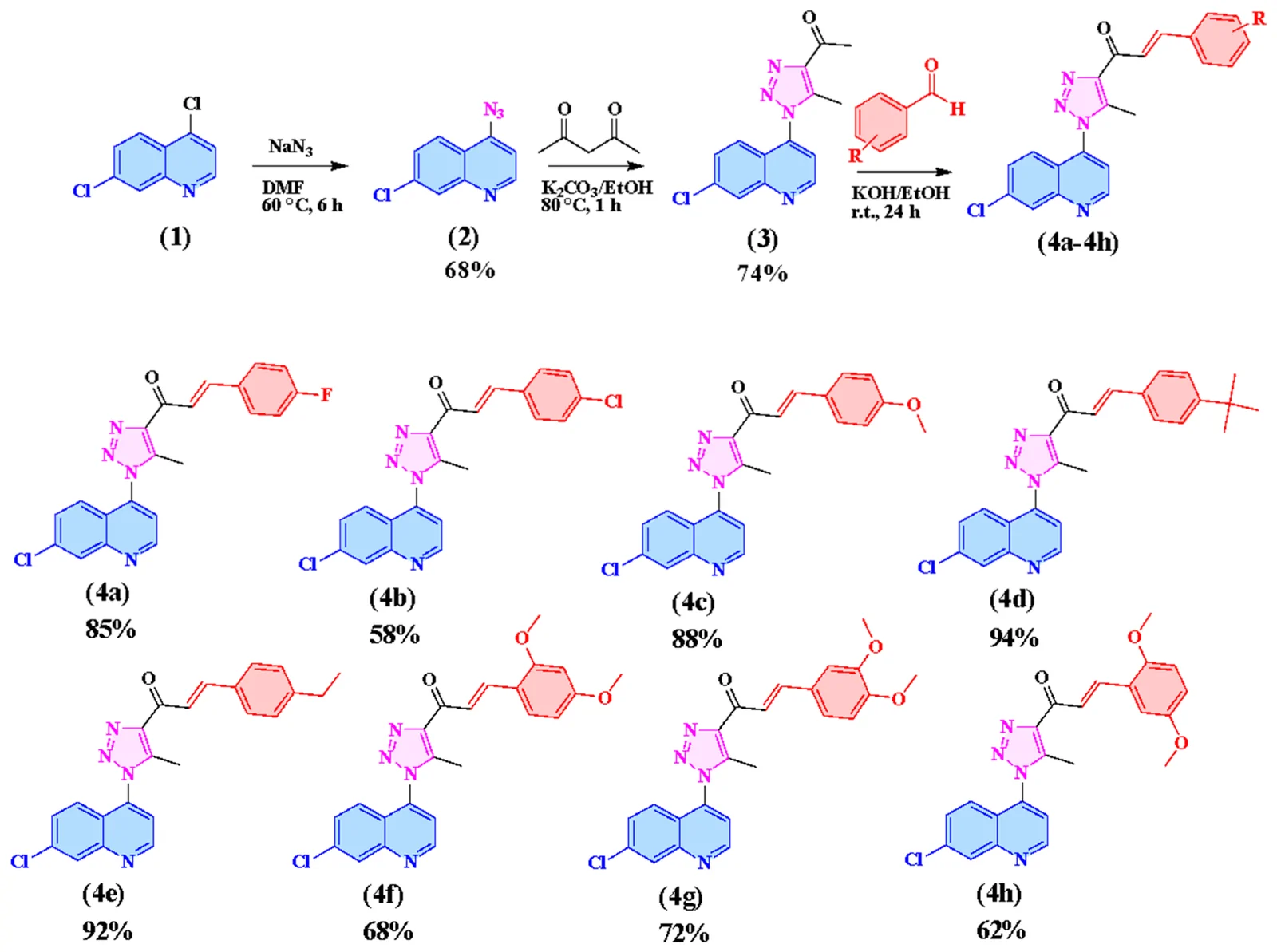
Total synthesis route for the novel (*E*)-1-(1-(7-chloroquinolin-4-yl)-5-methyl-1*H*-1,2,3-triazol-4-yl)-3-(*R*)prop-2-en-1-one (**4a**–**4h**).

The synthetic route to achieve the 1,2,3-triazole–quinoline derivatives is outlined in Scheme 1. The derivatives were prepared via a simple three-step route with high overall yields (58–94%). Initially, the commercially available starting material 4,7-dichloroquinoline (**1**) yielded intermediate 4-azido-7-chloroquinoline (**2**) via a nucleophilic aromatic substitution reaction with sodium azide (NaN_3_) in DMF. In the presence of K_2_CO_3_ as a base and EtOH as the solvent, intermediate **2** reacted with acetylacetone to obtain 1-(1-(7-chloroquinolin-4-yl)-5-methyl-1*H*-1,2,3-triazol-4-yl)ethan-1-one (**3**) via 1,3-dipolar cycloaddition. Subsequently, compound **3** reacted with various substituted benzaldehydes under basic conditions to yield the target products **4a**–**4h**. The newly synthesized compounds were fully characterized using physicochemical and spectroscopic measurements. Infrared (IR), nuclear magnetic resonance (NMR), and high-resolution mass spectrometry (HRMS) data are summarized in the Supplementary Materials and are fully consistent with the proposed molecular structures.

### 3.2. In Vitro Antiviral Validation of 1,2,3-Triazole–Quinolines

**Figure 3:**
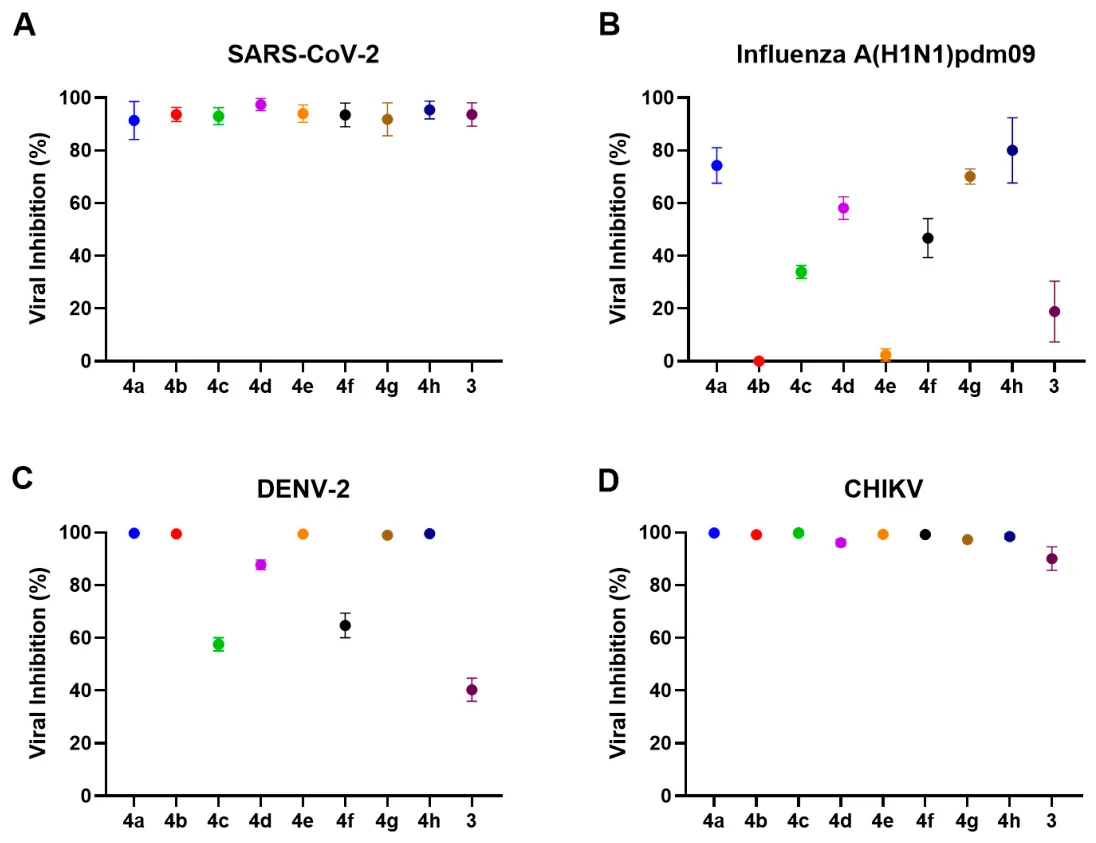
Initial antiviral screening for **3** and **4a**–**4h** at 10 µM. (**A**) Calu-3 cells infected with SARS-CoV-2, (**B**) MDCK with influenza A(H1N1)pdm09, and Huh-7 cells with (**C**) DENV-2 or (**D**) CHIKV. Infections were carried out at a multiplicity of infection (MOI) of 0.1 for 1 h at 37 °C. The inoculum was removed, the cells were washed and incubated with fresh DMEM containing 2% FBS, and the indicated concentrations of the compounds were added immediately thereafter. After 1 day for influenza or 2 days for the other viruses, supernatants were harvested and quantified. The data represent the mean  ±  SEMs of three independent experiments.

Antiviral screening for **4a**–**4h** was assessed at 10 µM against SARS-CoV-2, influenza A(H1N1)pdm09, DENV-2, and CHIKV (Figure 3). To evaluate the effect of incorporating the chalcone moiety into the 1-(1-(7-chloroquinolin-4-yl)-5-methyl-1*H*-1,2,3-triazol-4-yl)ethan-1-one core, intermediate **3** was also included in the biological assays. As depicted in Figure 3, all evaluated compounds exhibited high-percentage inhibition values at 10 µM against SARS-CoV-2 and CHIKV.

To confirm that the observed antiviral activity was not artifactual due to cytotoxicity, CC_50_ values were determined in Calu-3, MDCK, and Huh-7 (Table 1 and Table 2, Figures S1 and S2 in the Supplementary Materials). In all cases, CC_50_ values exceeded 10 µM; specifically, Calu-3 and MDCK cells displayed remarkably low cytotoxicity, with CC_50_ values exceeding 200 µM.

To better evaluate the antiviral efficacy of **3** and **4a**–**4h**, EC_50_ values against SARS-CoV-2, CHIKV, influenza A(H1N1)pdm09, and DENV-2 were determined (Table 1 and Table 2). The low EC_50_ values observed for **4a**–**4h** reflect potent inhibitory profiles. Notably, in most instances, coupling the chalcone motif to core **3** markedly decreased EC_50_ values—for example, yielding approximately 5-fold and 8-fold potency enhancements against SARS-CoV-2 replication for compounds **4c** and **4e**, respectively, and 6-fold and 29-fold enhancements against DENV-2 replication for the corresponding compounds. Although compound **4e** presented a lower CC_50_ (12 μM) in Huh-7 cells, it achieved 90% inhibition in primary screening against CHIKV and DENV-2, with dose–response curves revealing 9-fold and 45-fold safety margins between its EC_50_ and CC_50_ values, respectively.

Preliminary structure–activity relationship (SAR) analysis against SARS-CoV-2 (Table 1) revealed that derivatives bearing *para*-methoxy (**4c**) and *para*-ethyl (**4e**) groups on the phenyl ring exhibited the highest selectivity indices, greatest antiviral potency, and lowest cytotoxicity. Conversely, against influenza A(H1N1)pdm09 (Table 1), the safest and most potent derivatives were those featuring bulkier substituents—specifically 2,5-dimethoxy (**4h**), followed by *tert*-butyl (**4d**).

When comparing dimethoxy isomers (**4f**–**4h**) against SARS-CoV-2, the 2,5-dimethoxy derivative (**4h**) demonstrated 1.5-fold higher potency than the 2,4-dimethoxy (**4f**) and 3,4-dimethoxy (**4g**) analogs. Against influenza A(H1N1)pdm09, compounds **4f** and **4h** were approximately 4.5-fold more potent than **4g**.

For the evaluated arboviruses (Table 2), **4a**–**4h** displayed generally similar antiviral potencies against CHIKV, except for **4f**, which was 3.2- to 5.3-fold less potent than the rest of the series. Among the dimethoxylated chalcone isomers against CHIKV, activity was dependent on the substitution pattern: 2,5-dimethoxy (**4h**) exhibited ~5.3-fold and ~1.6-fold higher potency than 2,4-dimethoxy (**4f**) and 3,4-dimethoxy (**4g**), respectively.

### 3.3. In Silico Drug-likeness and Antiviral Prediction of 1,2,3-Triazole–Quinoline Derivatives

**Figure 4:**
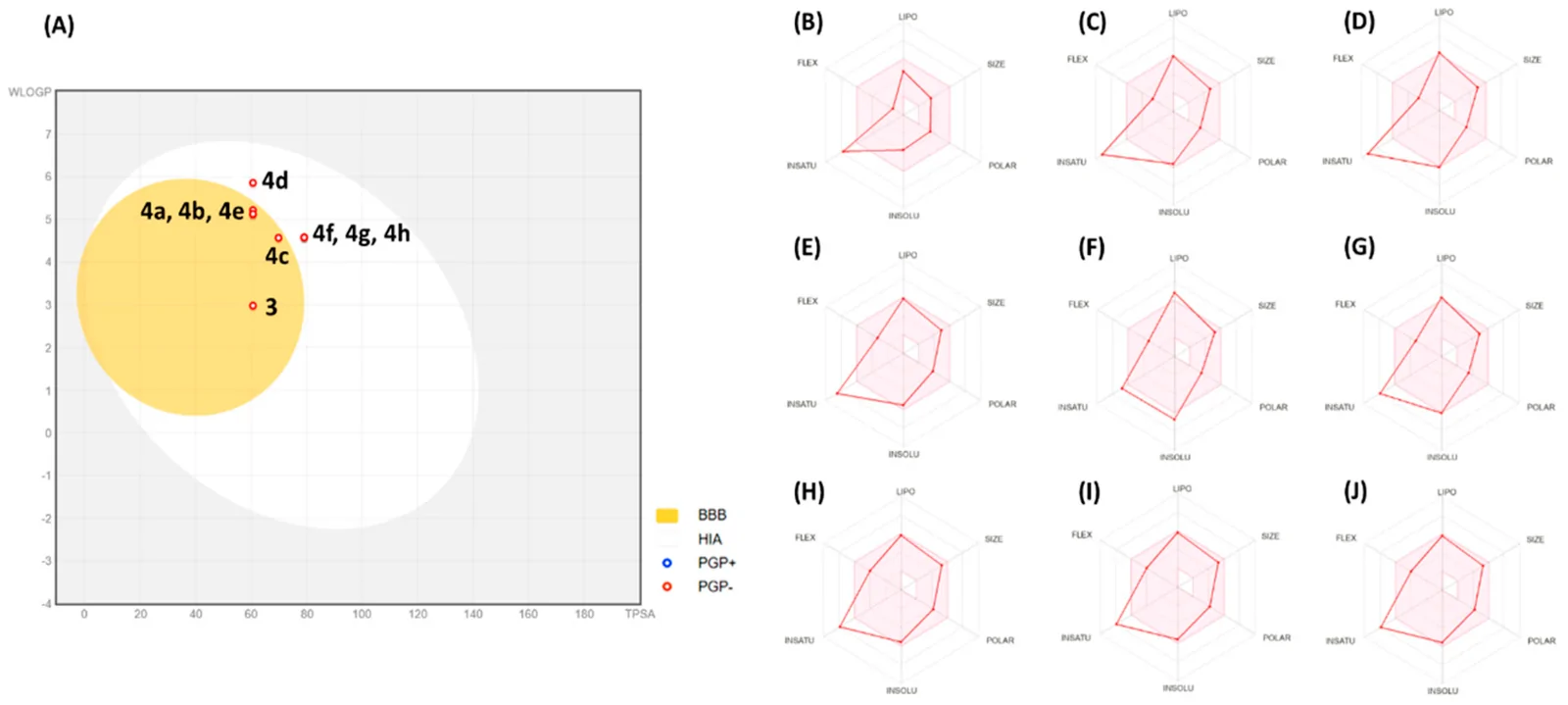
(**A**) The BOILED-Egg graphic for the 1,2,3-triazole–quinoline derivatives. The computed bioavailability radar for (**B**) **3**, (**C**) **4a**, (**D**) **4b**, (**E**) **4c**, (**F**) **4d**, (**G**) **4e**, (**H**) **4f**, (**I**) **4g**, and (**J**) **4h**. The HIA, BBB, PGP^+^, and PGP^−^ mean passive gastrointestinal absorption, blood–brain barrier permeation, P-glycoprotein1 substrate, and P-glycoprotein1 non-substrate, respectively.

In silico prediction of ADME parameters provides key preliminary insights into a candidate’s in vivo potential. ADME, physicochemical, and drug-likeness profiles for intermediate **3** and derivatives **4a**–**4h** were predicted and are summarized in Table 3. The brain or intestinal estimated permeation model (BOILED-Egg, Figure 4) indicated that appending the chalcone motif to the 1,2,3-triazole–quinoline core increases lipophilicity (raising iLOGP values from 2.62 up to 5.33, Table 3).

**Figure 5:**
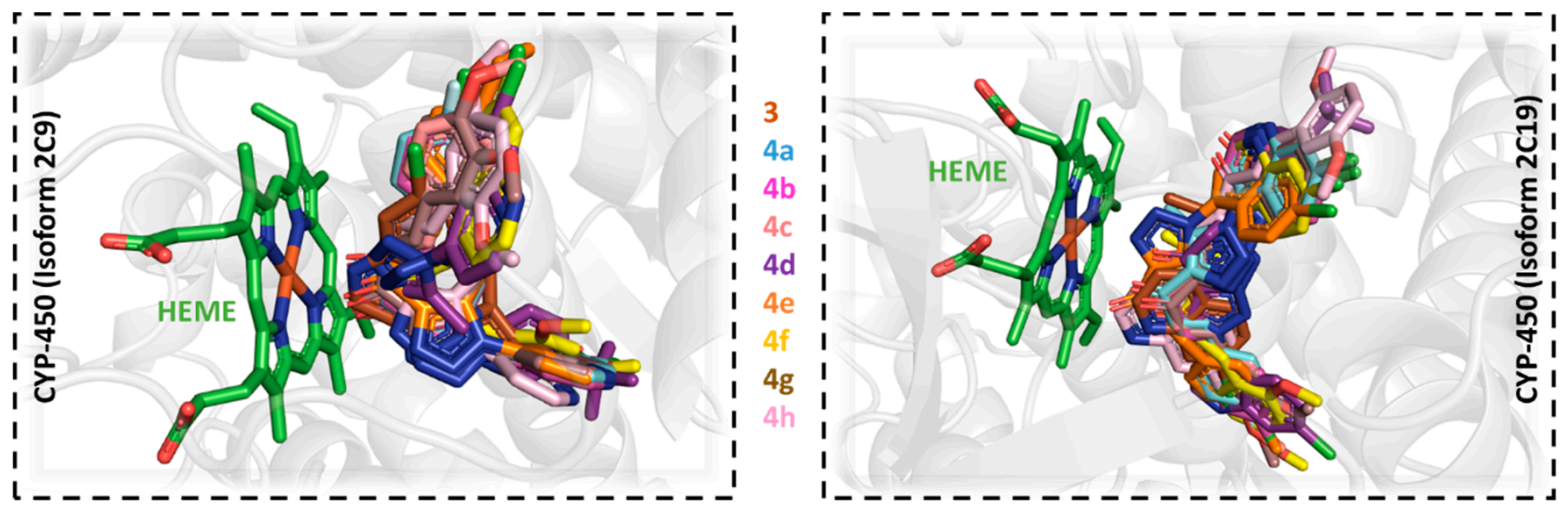
The best docking pose for the interaction between 1,2,3-triazole–quinoline derivatives and two isoforms of human CYP450 at the catalytic site of the enzyme. For better interpretation, hydrogen atoms were omitted. Elements’ colors: oxygen, nitrogen, fluorine, and chlorine are in red, dark blue, light blue, and dark green, respectively.

Computational modeling also predicted potential interactions between compounds **3** and **4a**–**4h** and major cytochrome P450 (CYP) isoforms, particularly CYP2C9 and CYP2C19 (Table 3). Compound **3** and derivatives **4a**–**4e** were predicted to interact with CYP1A2, CYP2C19, and CYP2C9, whereas compounds **4f**–**4h** showed interactions with CYP2C19, CYP2C9, and CYP3A4. The molecular docking analysis further indicated that the 1,2,3-triazole–quinoline derivatives could occupy the catalytic sites of the evaluated CYP450 isoforms (2C9 and 2C19, Figure 5).

Overall, the designed 1,2,3-triazole–quinoline derivatives—particularly those bearing the chalcone motif—exhibited promising drug-likeness, as illustrated by their bioavailability radar plots (Figure 4B–J) and confirmed by zero or minimal violations of major drug-likeness rules (Lipinski, Ghose, Veber, Egan, and Muegge; Table 3).

## 4. Discussion

Chloroquine is a 4-aminoquinoline antimalarial drug that is also recognized for its antiviral activity against several viral pathogens, including coronaviruses, flaviviruses, and retroviruses [30,31]. Its antiviral and immunomodulatory properties are associated with its ability to readily cross cellular membranes, thereby enabling interactions with intracellular targets and pathways involved in viral replication and host immune responses [31]. Furthermore, the chemical structure of chloroquine provides a suitable scaffold for the incorporation of additional pharmacologically active moieties, such as triazole derivatives, which may contribute to the enhancement or modulation of its biological activity [32]. In addition to the potential improvement of its antiviral properties, such structural modifications are particularly relevant from a pharmaceutical perspective, as chloroquine is an established antimalarial agent with a well-established manufacturing infrastructure and sustained global demand. Consequently, the development of structurally modified chloroquine scaffold hybrids may represent a promising strategy for expanding its therapeutic applications while leveraging the existing availability and production of the parent compound.

The antiviral activity observed for **3** and **4a–4h** showed that incorporation of the chalcone moiety into the 1,2,3-triazole–7-chloroquinoline scaffold generally improved the antiviral profile, although marked differences in inhibition were observed among the evaluated viruses. Against SARS-CoV-2, incorporation of the chalcone motif increased the potency of **4c** and **4e** relative to core structure **3**, whereas activity against influenza A(H1N1)pdm09 and CHIKV showed greater dependence on the chemical substituents on the aromatic ring of the chalcone moiety. These findings are consistent with previous studies showing that structural modification of quinoline-based scaffolds can influence antiviral activity. Boechat et al. [33] reported 1,2,3-triazolyl-4-oxoquinoline derivatives with antiviral activity against influenza A and B viruses, while Forezi et al. [34] showed that structural modifications of 4-oxoquinoline derivatives influenced their antiviral activity. Barbosa-Lima et al. [14] also reported improved antiviral activity of structurally modified quinoline derivatives against Zika virus. Together, these studies support the use of quinoline-containing scaffolds as a platform for the development of structurally modified antiviral compounds.

The differences in cytotoxicity observed among the evaluated cell lines are consistent with previous reports involving related quinoline-containing compounds. Rachakonda et al. [35] previously identified quinolinyl-pyrazole hybrids with low cytotoxicity toward lung epithelial cancer cells (A549), which varied depending on the pyrazole substituent. Similarly, Awolade et al. [36] reported quinoline–1,2,3-triazole hybrids with low cytotoxicity against human embryonic kidney cells (HEK293), and Bhukya et al. [37] demonstrated cell-line-dependent differences in CC_50_ values for quinoline-linked triazoles across A549, breast cancer (MCF-7), and liver cancer (HepG2) lines. These reports support the cell-type-dependent cytotoxicity variations observed in our dataset.

Consequently, the selectivity indices also differed among the synthesized derivatives (Table 1 and Table 2). Against SARS-CoV-2, **4c** and **4e** displayed the highest SI values within the series, with **4e** exceeding the SI of RDV. On the other hand, **4h** presented the best SI for influenza, followed by **4g**. Although data for **3** and **4a–4h** against DENV-2 and CHIKV showed higher cytotoxicity in Huh-7 cells than in Calu-3 or MDCK cells, incorporation of the chalcone motif generally enhanced antiviral activity against the arboviruses, resulting in improved selectivity indices. This pattern, together with the differences observed among the compounds against the evaluated viruses, indicates a lack of direct SAR correlation between the viral families. Previous studies of quinoline derivatives have likewise reported differences in antiviral activity and selectivity depending on the structural modifications introduced into the quinoline scaffold [14,33].

This differential activity suggests that **4f**–**4h** may interact differently with viral or host targets, which aligns with published literature identifying quinoline derivatives as multi-target agents capable of blocking viral entry as well as replication [13,38,39]. For instance, Sabt et al. [40] reported 1-aryl-1,2,3-triazole–quinoline hybrids functioning as neuraminidase inhibitors, while Li et al. [41] described quinoline derivatives containing piperazine rings as inhibitors of viral RNA transcription and replication. Although the present results do not establish the specific molecular targets responsible for the observed differences, these reports provide a possible context for the distinct antiviral profiles observed among **4f**–**4h**. The substitution pattern also appears to influence antiviral activity against the evaluated arboviruses. Simultaneously, *ortho*- and *para*-methoxy substitution (**4f**) may impair antiviral activity against CHIKV, while a similar trend was observed among **4f**–**4h** against DENV-2. These differences suggest that the antiviral profile of the dimethoxy derivatives is influenced not only by the presence of methoxy groups but also by their position within the aromatic ring. This type of structure-dependent activity is consistent with the findings of Forezi et al. [34], in which structural and conformational features influenced the antiviral activity of 4-oxoquinoline derivatives.

The in silico analyses provide additional insight into the physicochemical and pharmacokinetic characteristics of the synthesized compounds. This increased lipophilicity likely enhances cellular uptake, providing a plausible mechanism for the improved antiviral potency of **4a–4h** over core **3** [42]. Furthermore, methoxy substituents on the aromatic ring increased the topological polar surface area (TPSA, Table 3) into an optimal range for passive gastrointestinal absorption. All 1,2,3-triazole–quinoline derivatives were predicted to be non-substrates for P-glycoprotein (P-gp), which may prevent active efflux and increase intracellular accumulation [43]. These predicted properties provide additional support for the favorable drug-like profile of the synthesized derivatives.

Computational modeling also predicted potential interactions between compounds **3** and **4a**–**4h** and CYP450 isoforms, particularly CYP2C9 and CYP2C19 (Table 3). The predicted interaction involving the 1,2,3-triazole core and the Fe(II) center of the heme group (Figure 5) [44] may provide a possible explanation for the higher experimental cytotoxicity observed in Huh-7 cells compared to Calu-3 and MDCK cells. However, this relationship remains a predicted association and requires experimental validation. Overall, the profile was further supported by zero or minimal violations of major drug-likeness rules (Lipinski, Ghose, Veber, Egan, and Muegge; Table 3) [45].

In general, the above-described results support the molecular hybridization strategy, showing that integrating the quinoline, 1,2,3-triazole, and chalcone pharmacophores yields functional derivatives with antiviral activity against different viral families. The combined experimental and in silico results indicate that the biological activity of these derivatives is influenced by the chemical substituents incorporated into the chalcone moiety and their position on the aromatic ring, while their predicted physicochemical and drug-likeness properties support their further investigation as antiviral prototypes.

## 5. Conclusions

A concise and efficient synthetic strategy was developed to access a novel series of 1,2,3-triazole–quinoline derivatives (**4a**–**4h**) in moderate-to-high yields. The synthesized compounds were subsequently evaluated in vitro against SARS-CoV-2, influenza A(H1N1)pdm09, DENV-2, and CHIKV, as well as for cytotoxicity. The incorporation of the chalcone motif in the chemical structure of **3** increased the biological activity, acting as a feasible broad-spectrum antiviral with positive predicted drug-likeness properties. Notably, compounds **4e** and **4c** exhibited outstanding selectivity indices of 1896.5 and 1265.8, respectively, against SARS-CoV-2 replication, highlighting their potential as promising antiviral candidates.

## Figures and Tables

**Table 1:** Antiviral parameters against SARS-CoV-2 and influenza A(H1N1)pdm09, with the corresponding cytotoxicity values in Calu-3 and MDCK cells.

Compounds	Calu-3 CC_50_ (µM) (SD)	SARS-CoV-2 EC_50_ (µM) (SD)	SI *	MDCK CC_50_ (µM)	Influenza EC_50_ (µM) (SD)	SI *
**3**	>1000	4.0 (0.20)	250	>500	ND **	ND **
**4a**	>1000	2.0 (0.10)	500	>500	9.48 (0.47)	52.7
**4b**	>1000	3.0 (0.15)	333.33	376.5 (24.1)	ND	ND
**4c**	>1000	0.8 (0.04)	1265.80	>500	10.35 (0.52)	48.3
**4d**	>1000	5.0 (0.25)	200.0	18.6 (7.7)	0.96 (0.05)	19.4
**4e**	948 (75)	0.5 (0.03)	1896.50	388.5 (13.9)	ND **	ND **
**4f**	>1000	3.0 (0.15)	333.33	337.3 (21.3)	1.23 (0.06)	274.2
**4g**	683 (23)	3.0 (0.15)	227.85	>500	5.96 (0.30)	83.9
**4h**	912 (18)	2.0 (0.10)	456.23	>500	1.37 (0.07)	364.9
**Chloroquine**	98 (16)	>10	—	ND	ND	ND
**Remdesivir**	317 (23)	0.2 (0.01)	1585	ND	ND	ND
**Oseltamivir**	ND	ND	ND	>500	0.14 (0.09)	3571.4

Data are presented as mean ± SEMs from three independent experiments. SI *—selectivity index; ND **—not determined.

**Table 2:** Antiviral parameters against arboviruses CHIKV and DENV-2 with the corresponding cytotoxicity values in Huh-7 cells.

Compounds	Huh-7 CC_50_ (µM) (SD)	CHIKV EC_50_ (µM) (SD)	SI *	DENV-2 EC_50_ (µM) (SD)	SI *
**3**	43 (3.6)	ND **	ND **	12.8 (8.5)	3.4
**4a**	37 (8.7)	1.4 (0.07)	25.98	3.1 (0.4)	11.9
**4b**	37 (2.3)	1.2 (0.06)	32.15	5.8 (1.9)	6.4
**4c**	46 (1.4)	1.2 (0.06)	37.53	ND **	ND **
**4d**	27 (2.2)	1.2 (0.06)	22.11	0.8 (0.1)	33.7
**4e**	12 (1.7)	1.5 (0.07)	8.93	1.7 (0.2)	7
**4f**	37 (5.4)	4.8 (0.24)	7.86	ND **	ND **
**4g**	34 (2.3)	1.4 (0.07)	25.01	3.5 (0.5)	9.7
**4h**	43 (1.9)	0.9 (0.04)	49.13	4.3 (0.4)	10
**Chloroquine**	58 (12.0)	2.4 (0.80)	24.17	5.4 (0.90)	10.10
**Remdesivir**	128 (24.0)	>10	—	0.8 (0.03)	160.00

Data are presented as mean ± SEMs from three independent experiments. SI *—selectivity index; ND **—not determined.

**Table 3:** Selected physicochemical, drug-likeness, and pharmacokinetic properties were predicted by the free web server SwissADME.

Compound	Consensus Log *P_o/w_*	CYP Interaction Isoform		Drug-Likeness (Number of Violations)				
			TPSA (Å^2^)	Lipinski	Ghose	Veber	Egan	Muegge
**3**	2.62	1A2, 2C19, 2C9	60.67	Yes (0)	Yes (0)	Yes (0)	Yes (0)	Yes (0)
**4a**	4.44	1A2, 2C19, 2C9	60.67	Yes (0)	Yes (0)	Yes (0)	Yes (0)	Yes (0)
**4b**	4.65	1A2, 2C19, 2C9	60.67	Yes (0)	Yes (0)	Yes (0)	Yes (0)	No (1)
**4c**	4.12	1A2, 2C19, 2C9	69.90	Yes (0)	Yes (0)	Yes (0)	Yes (0)	Yes (0)
**4d**	5.33	2C19, 2C9	60.67	Yes (0)	No (1)	Yes (0)	Yes (0)	No (1)
**4e**	4.76	1A2, 2C19, 2C9	60.67	Yes (0)	Yes (0)	Yes (0)	Yes (0)	No (1)
**4f**	4.13	2C19, 2C9, 3A4	79.13	Yes (0)	Yes (0)	Yes (0)	Yes (0)	Yes (0)
**4g**	4.11	2C19, 2C9, 3A4	79.13	Yes (0)	Yes (0)	Yes (0)	Yes (0)	Yes (0)
**4h**	4.12	2C19, 2C9, 3A4	79.13	Yes (0)	Yes (0)	Yes (0)	Yes (0)	Yes (0)

## Data Availability

Data are contained within the article or Supplementary Materials.

## Supplementary Materials

The following supporting information can be downloaded at: https://www.mdpi.com/article/10.3390/v18090951/s1, File S1: IR, ^1^H NMR, and ^13^C NMR of compounds **2**, **3**, and **4a**–**4h**; SMILES strings for **3** and **4a**–**4h**; Figure S1: Dose-response curves and viability in Huh-7 cells; Figure S2: Dose-response curves and viability in MDCK and Calu-3 cells.
