# Supplementary material for: Synthesis and Broad-Spectrum Antiviral Evaluation of Novel 1,2,3-Triazole–Quinoline Derivatives Targeting SARS-CoV-2, Influenza, DENV-2, and CHIKV
Source: Viruses. 2026 Aug 31;18(9):951. doi: 10.3390/v18090951 (PMC13611932; doi:10.3390/v18090951)
Supplement: Supplementary file 1 [file viruses-18-00951-s001.zip › viruses-4506024-supplementary.pdf]

SUPPLEMENTARY MATERIAL

Synthesis and Broad-Spectrum Antiviral Evaluation of Novel 1,2,3-Triazole–Quinoline Derivatives Targeting SARS-CoV-2, Influenza, DENV-2, and CHIKV

Alcione Silva de Carvalho [1,†], Natalia Fintelman-Rodrigues [2,3,†], Victória Toledo Diniz de Camargo [1], Lina Silva-Trujillo [2,3], Caroline de Souza de Freitas [2,3,4], Isabela Alencar Graciano [1], Acácio Silva de Souza [1], Camilla Blanco de Assis [2,3], Otávio Augusto Chaves [2,3,5], Fernando de Carvalho da Silva [6], Vitor F. Ferreira [1,*] and Thiago Moreno L. Souza [2,3,7,*]

[1] Programa de Pós-Graduação em Ciências Aplicadas a Produtos para a Saúde, Faculdade de Farmácia, Universidade Federal Fluminense, Niterói 24241-000, RJ, Brazil

[2] Laboratory of Immunopharmacology, Center for Research, Innovation, and Surveillance in COVID-19 and Health Emergencies, Oswaldo Cruz Institute, Rio de Janeiro 21040-361, RJ, Brazil

[3] Center for Technological Development in Health, National Institute for Science and Technology on Innovation on Neglected Diseases and Neglected Populations, Oswaldo Cruz Foundation, Rio de Janeiro 21040-900, RJ, Brazil

[4] Laboratory of Viral Diseases Immunology, René Rachou Institute, Oswaldo Cruz Foundation, Belo Horizonte 30190-002, MG, Brazil

[5] Applied Molecular Chemistry Laboratory (LaQMA), Department of Chemistry and Environmental Sciences, Institute of Biosciences, Humanities and Exact Sciences (IBILCE), São Paulo State University (UNESP), São José do Rio Preto 15054-000, SP, Brazil

[6] Departamento de Química Orgânica, Instituto de Química, Universidade Federal Fluminense, Niterói 24020-141, RJ, Brazil

[7] Graduation Program on Surveillance in Health, Iguaçú University, Nova Iguaçu 26260-045, RJ, Brazil

* Correspondence: vitorferreira@id.uff.br (V.F.F.); thiago.moreno@fiocruz.br (T.M.L.S.)
† These authors contributed equally to this work.

.

INDEX

File S1

IR, ^{1}H NMR, and ^{13}C NMR of the compound **2** …………………………………… 3-5

IR, ^{1}H NMR, and ^{13}C NMR of the compound **3** ……………….…………… 6-8

IR, ^{1}H NMR, ^{13}C NMR, and HRMS of compound **4a** …………………….. 9-12

IR, ^{1}H NMR, ^{13}C NMR, and HRMS of compound **4b** …………………….. 13-16

IR, ^{1}H NMR, ^{13}C NMR, and HRMS of compound **4c** …………………….. 17-20

IR, ^{1}H NMR, ^{13}C NMR, and HRMS of compound **4d** …………………….. 21-24

IR, ^{1}H NMR, ^{13}C NMR, and HRMS of compound **4e** ……………........... 25-28

IR,^{1}H NMR, ^{13}C NMR, and HRMS of compound **4f** …………………… 29-32

IR,^{1}H NMR, ^{13}C NMR, and HRMS of compound **4g** …………………… 33-36

IR,^{1}H NMR, ^{13}C NMR, and HRMS of compound **4h** ……………........... 37-40

SMILES strings for **3**, **4a-4h** 41

Fig S1. Dose-response curves and viability in Huh-7 cells. Huh-7 cells were infected with **(A)** CHIKV or **(B)** DENV-2 and treated with concentrations ranging from 1.25 to 10 µM of the molecules**.** Cell viability was also evaluated **(C)** after adding different concentrations of the compounds. 42

Fig S2. Dose-response curves and viability in MDCK and Calu-3 cells. MDCK cells were infected with **(A)** Influenza A (H1N1pdm09), while Calu-3 cells were infected with **(B)** SARS-CoV-2; in both assays, cells were treated with concentrations ranging from 1.25 to 10 µM of the molecules**.** Cell viability was also evaluated in MDCK cells **(C)** and Calu-3 cells **(D)** after adding different concentrations of the compounds. 43

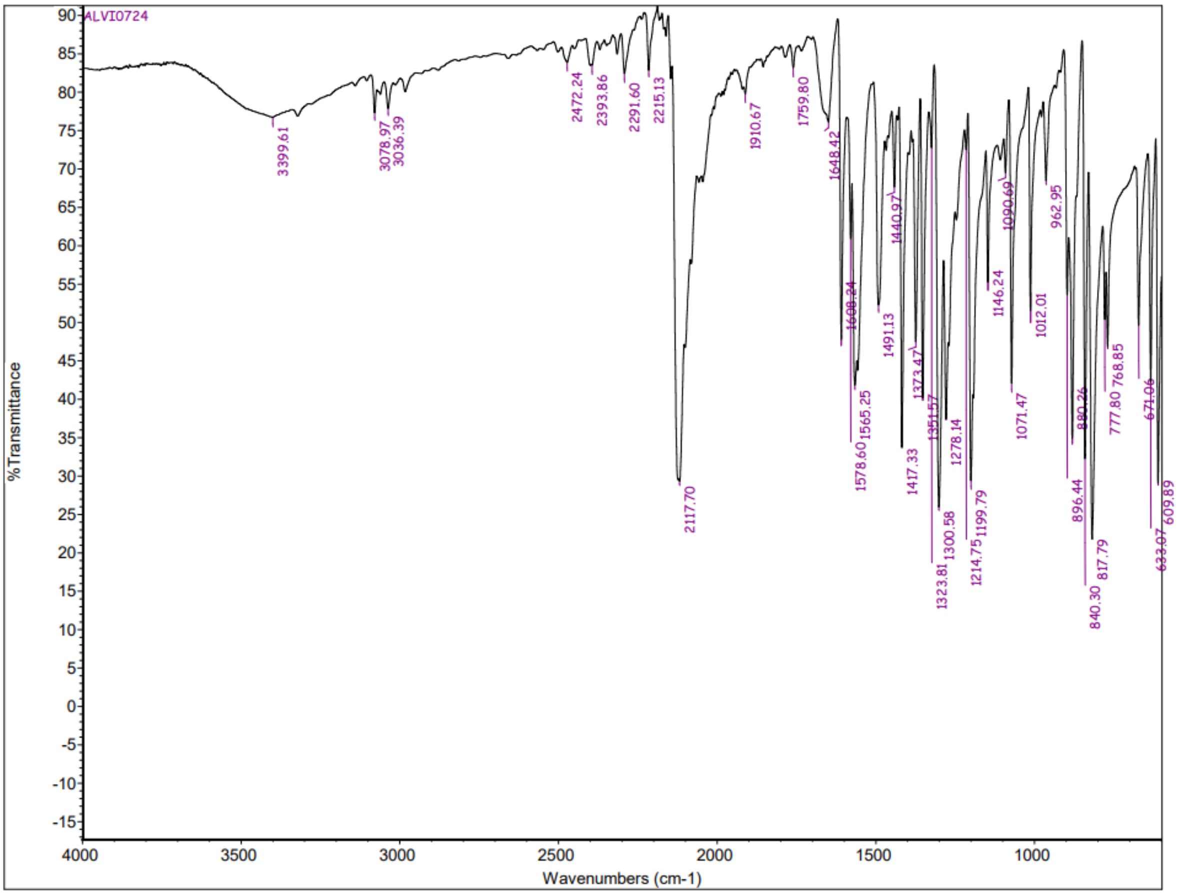

INFRARED SPECTRUM OF 4-AZIDOQUINOLINE **(2)**.

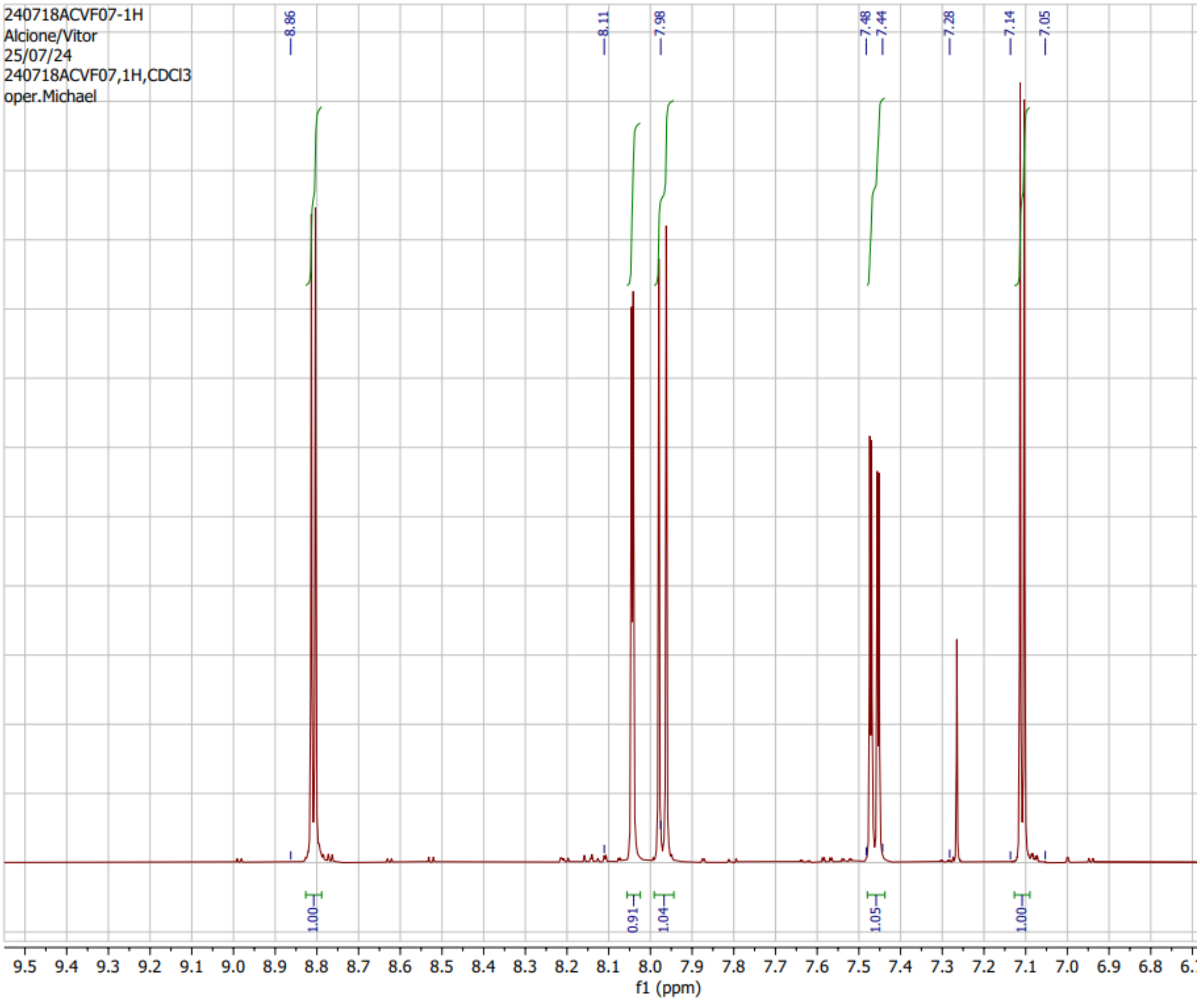

^{1}H NMR SPECTRUM OF 4-AZIDOQUINOLINE **(2)**.

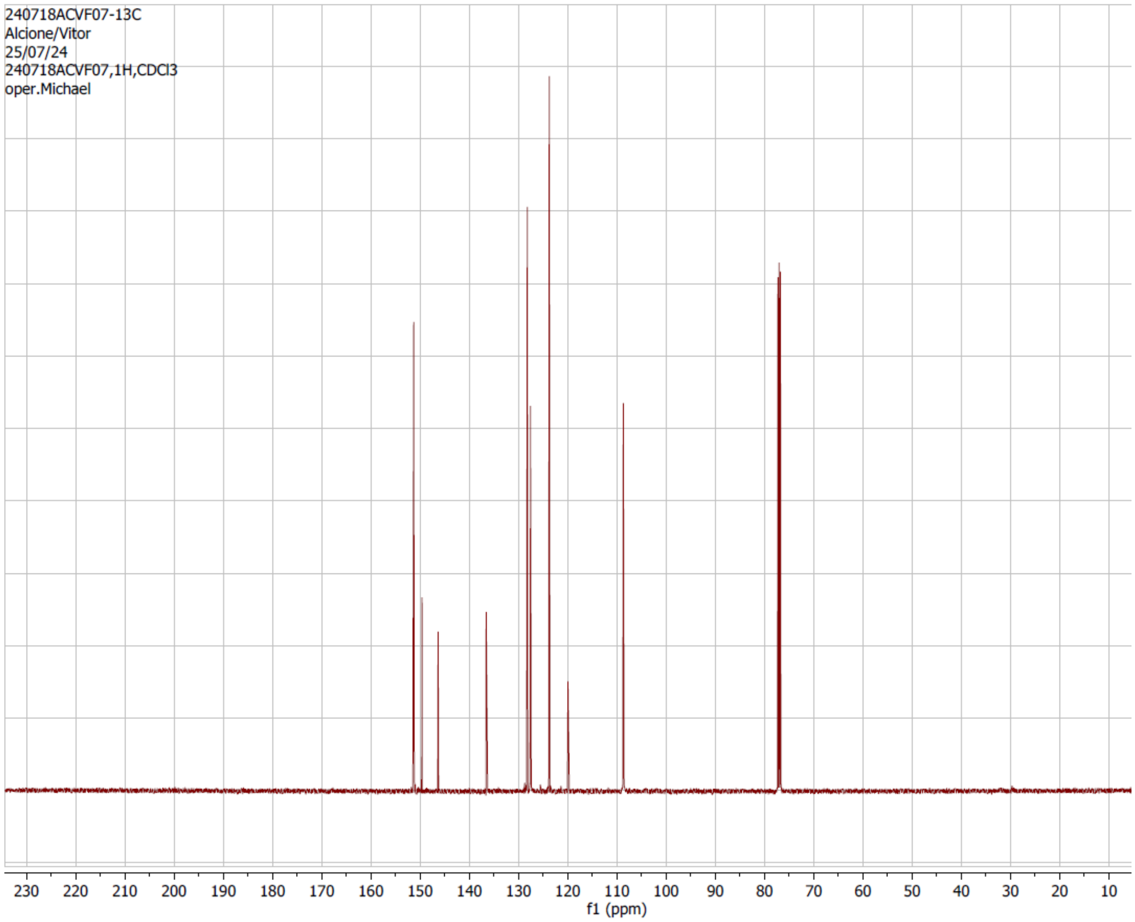

^{13}C NMR SPECTRUM OF 4-AZIDOQUINOLINE **(2)**.

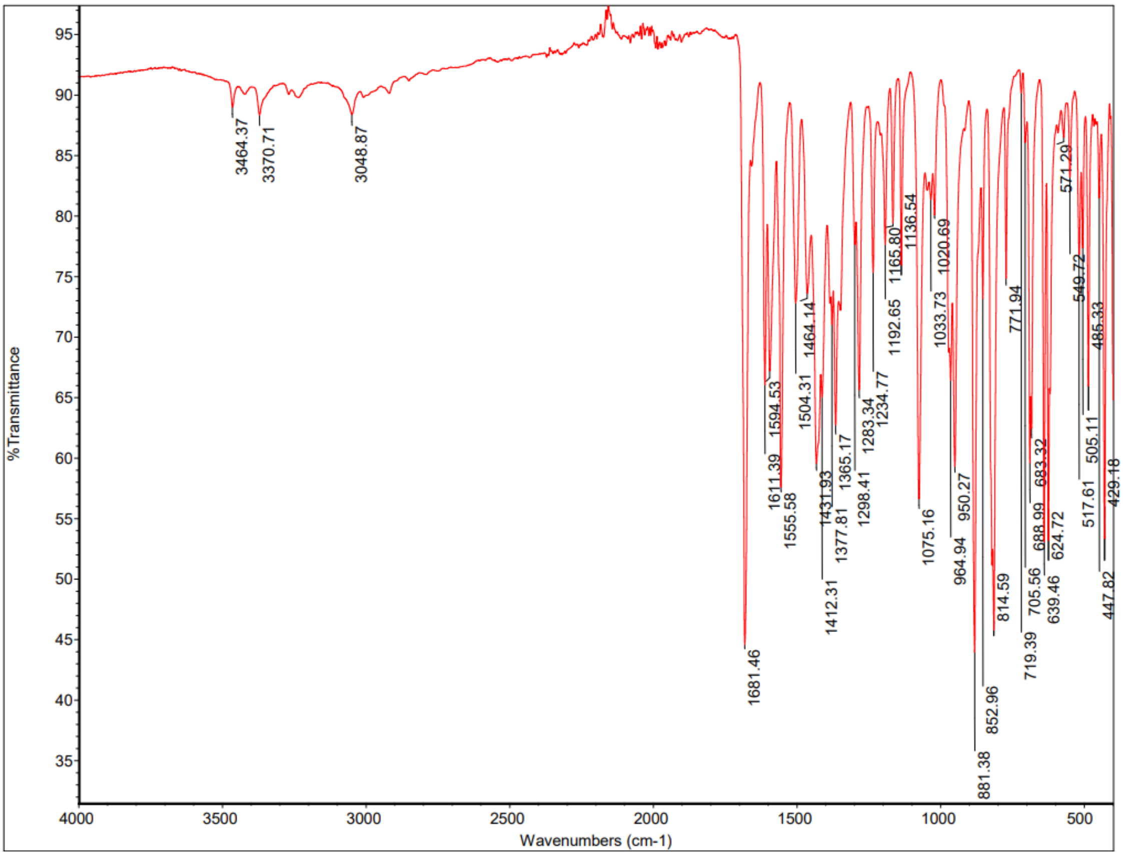

INFRARED SPECTRUM OF 1-(5-METHYL-1-(QUINOLIN-4-YL)-1*H*-1,2,3-TRIAZOL-4-YL)ETHAN-1-ONE **(3)**.

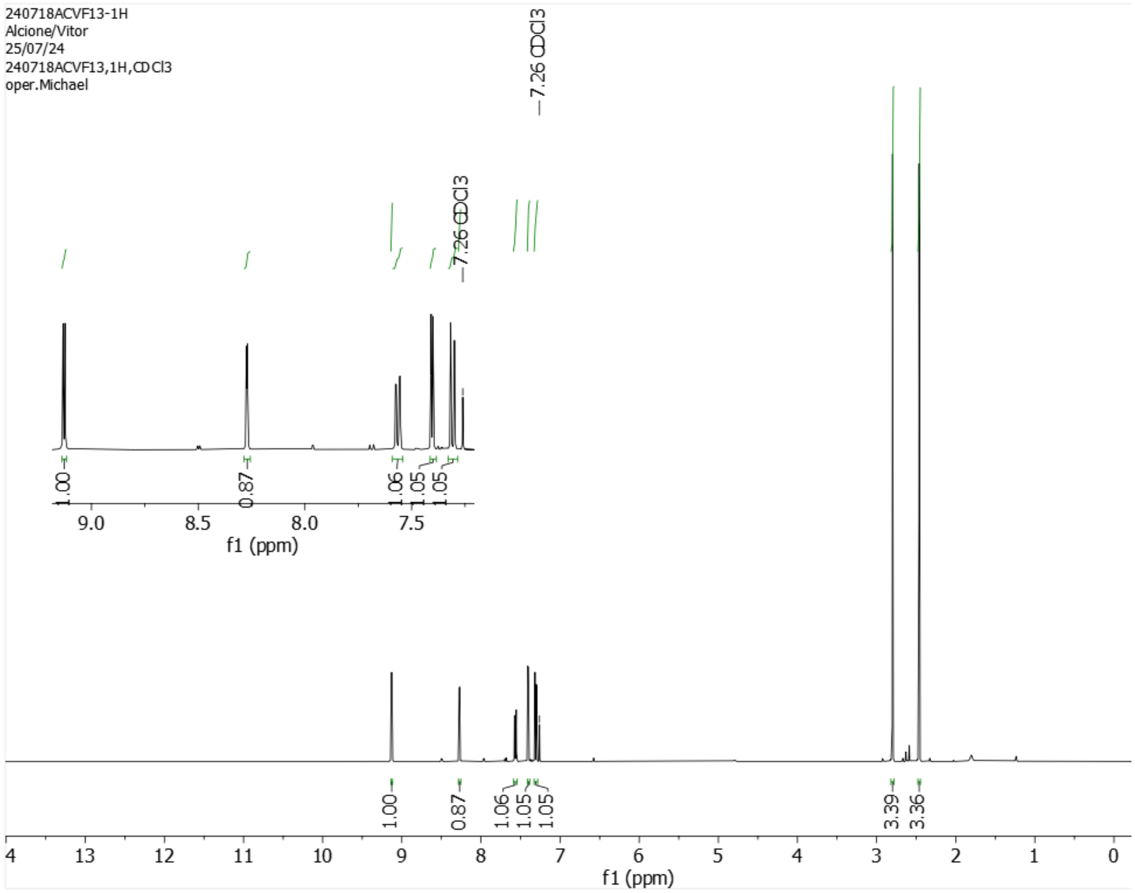

^{1}H NMR SPECTRUM OF 1-(5-METHYL-1-(QUINOLIN-4-YL)-1*H*-1,2,3-TRIAZOL-4-YL)ETHAN-1-ONE **(3)**.

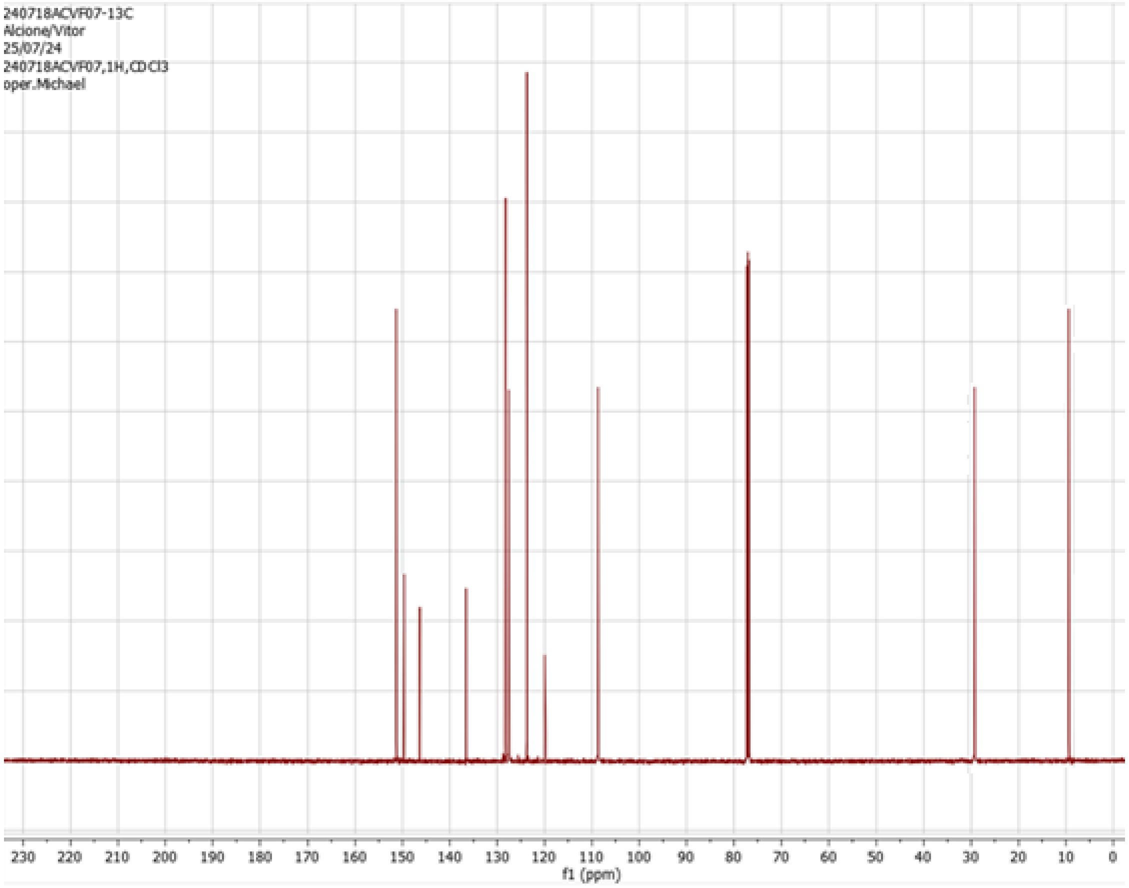

^{13}C NMR SPECTRUM OF 1-(5-METHYL-1-(QUINOLIN-4-YL)-1*H*-1,2,3-TRIAZOL-4-YL)ETHAN-1-ONE **(3)**.

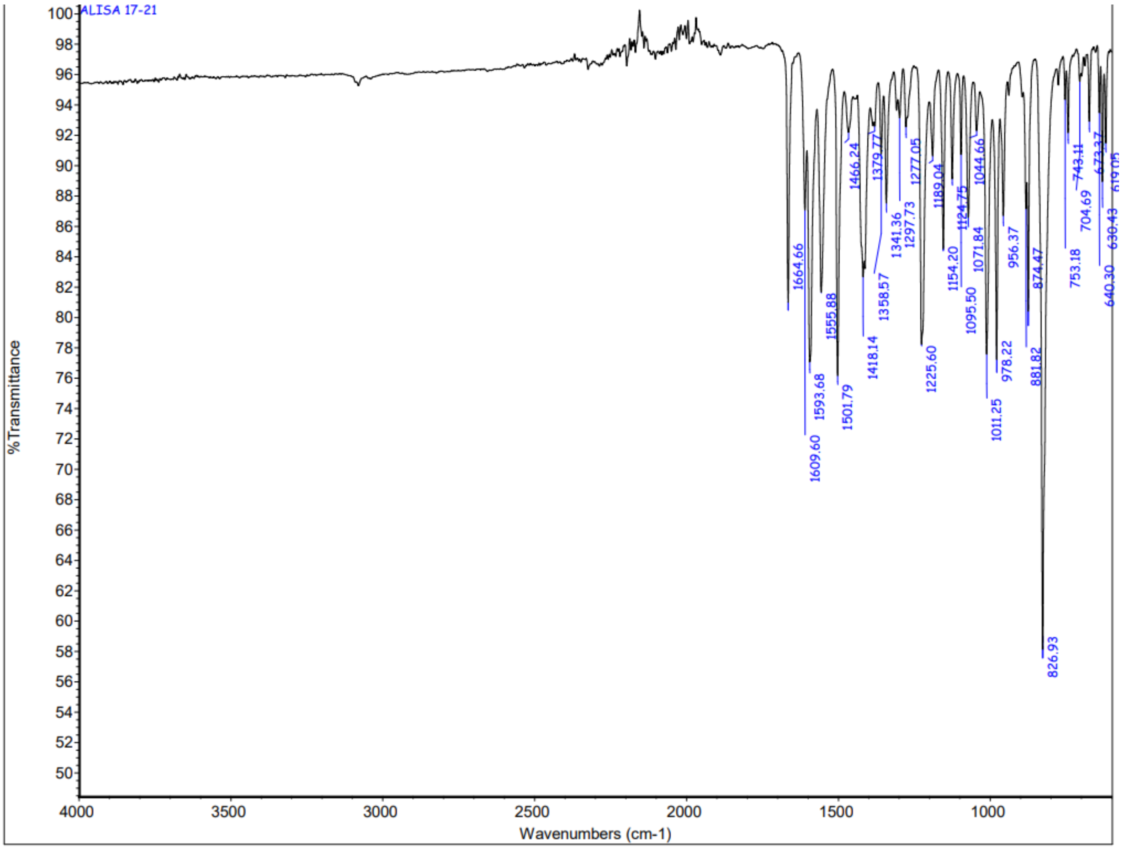

INFRARED SPECTRUM OF (*E*)-1-(1-(7-CHLOROQUINOLIN-4-YL)-5-METHYL-1*H*-1,2,3-TRIAZOL-4-YL)-3-(4-FLUOROPHENYL) PROP-2-EN-1-ONE **(4a)**.

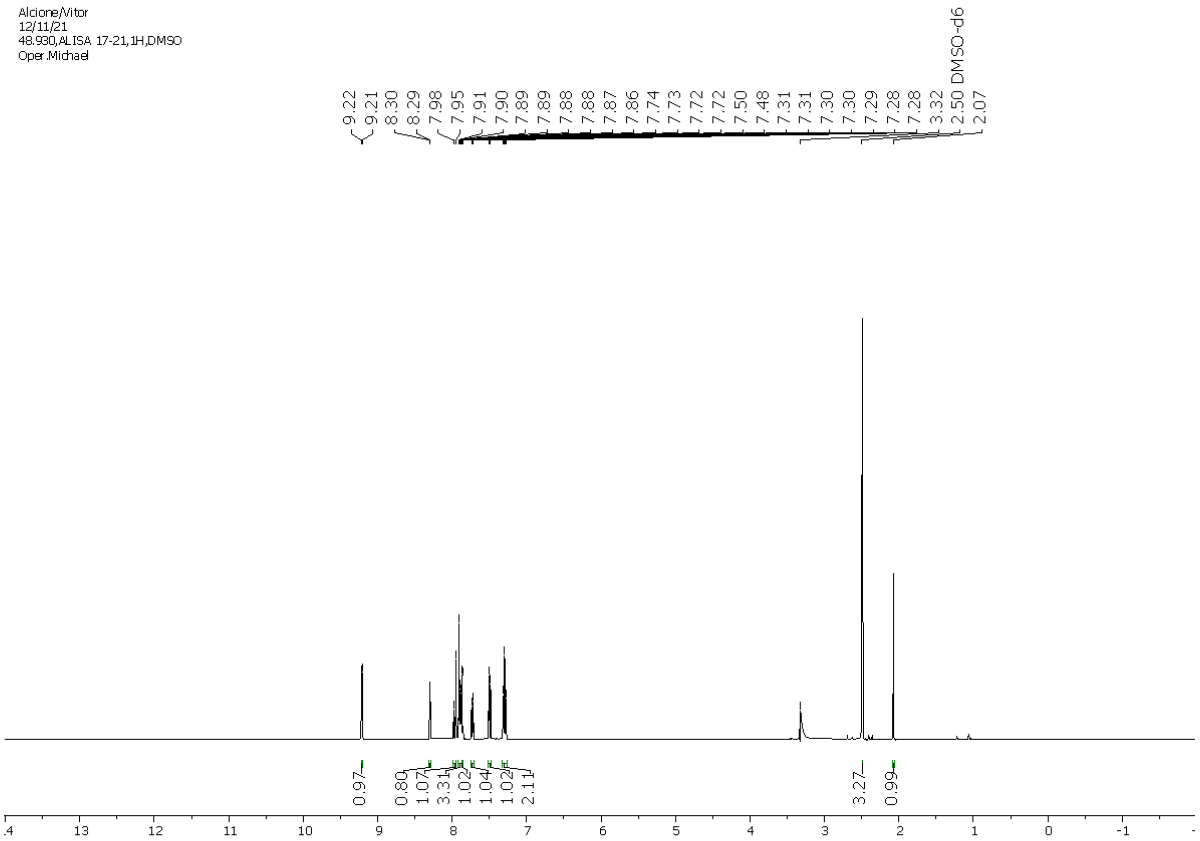

^{1}H-NMR SPECTRUM OF *E*)-1-(1-(7-CHLOROQUINOLIN-4-YL)-5-METHYL-1*H*-1,2,3-TRIAZOL-4-YL)-3-(4-FLUOROPHENYL) PROP-2-EN-1-ONE **(4a)**.

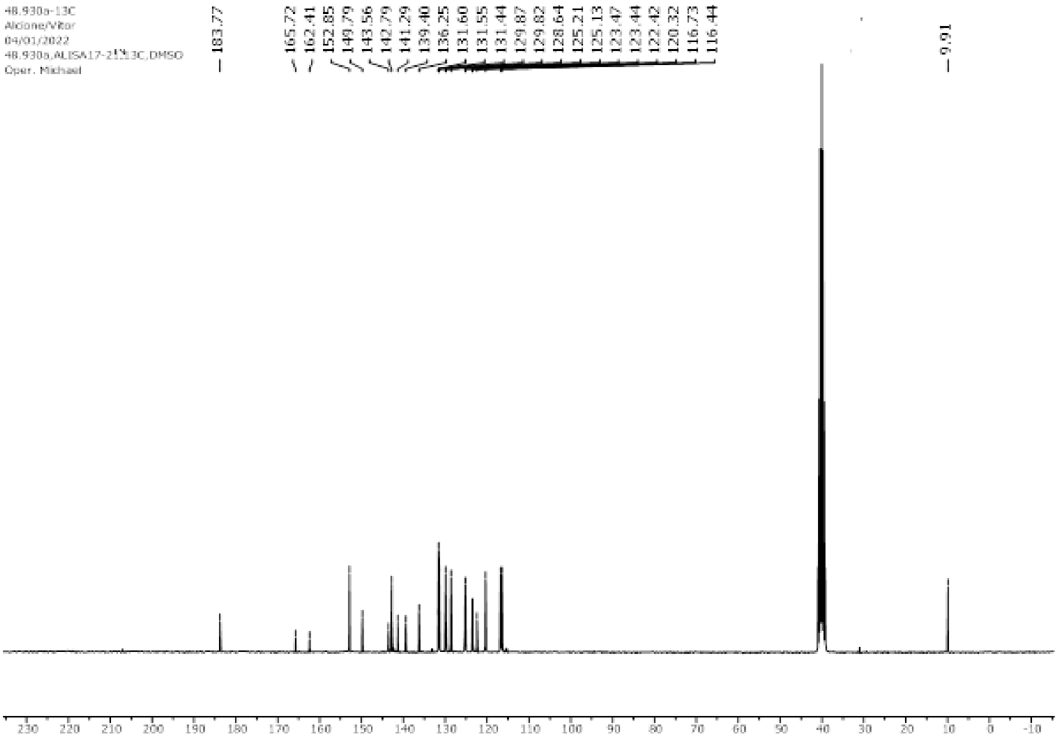

^{13}C-NMR SPECTRUM OF (*E*)-1-(1-(7-CHLOROQUINOLIN-4-YL)-5-METHYL-1*H*-1,2,3-TRIAZOL-4-YL)-3-(4-FLUOROPHENYL) PROP-2-EN-1-ONE **(4a)**.

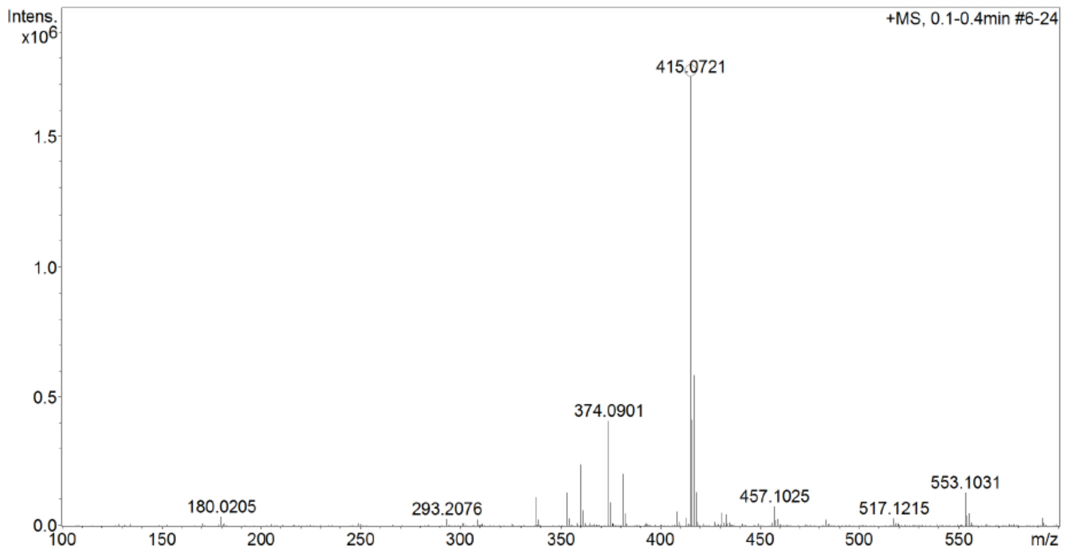

Meas. m/z	#	Ion Formula	m/z	err [ppm]	Mean err [ppm]	rdb	N-Rule	e⁻ Conf	mSigma	Std I	Std Mean m/z	Std I VarNorm	Std m/z Diff	Std Comb Dev
415.072066	1	C21H14ClFN4NaO	415.073238	2.8	2.6	15.5	ok	even	6.1	9.5	n.a.	n.a.	n.a.	n.a.

HRMS OF (*E*)-1-(1-(7-CHLOROQUINOLIN-4-YL)-5-METHYL-1*H*-1,2,3-TRIAZOL-4-YL)-3-(4-FLUOROPHENYL) PROP-2-EN-1-ONE **(4a)**.

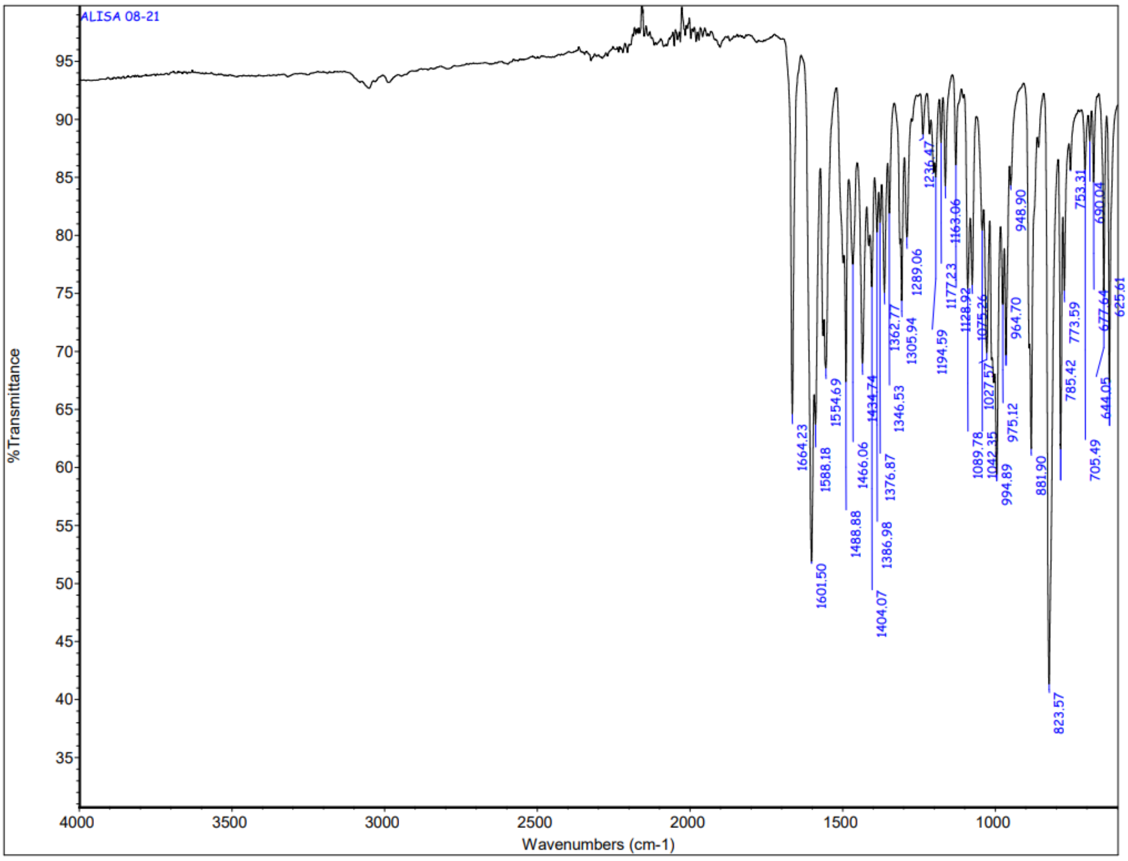

INFRARED SPECTRUM OF (*E*)-3-(4-CHLOROPHENYL)-1-(1-(7-CHLOROQUINOLIN-4-YL)-5-METHYL-1*H*-1,2,3-TRIAZOL-4-YL)PROP-2-EN-1-ONE **(4b)**.

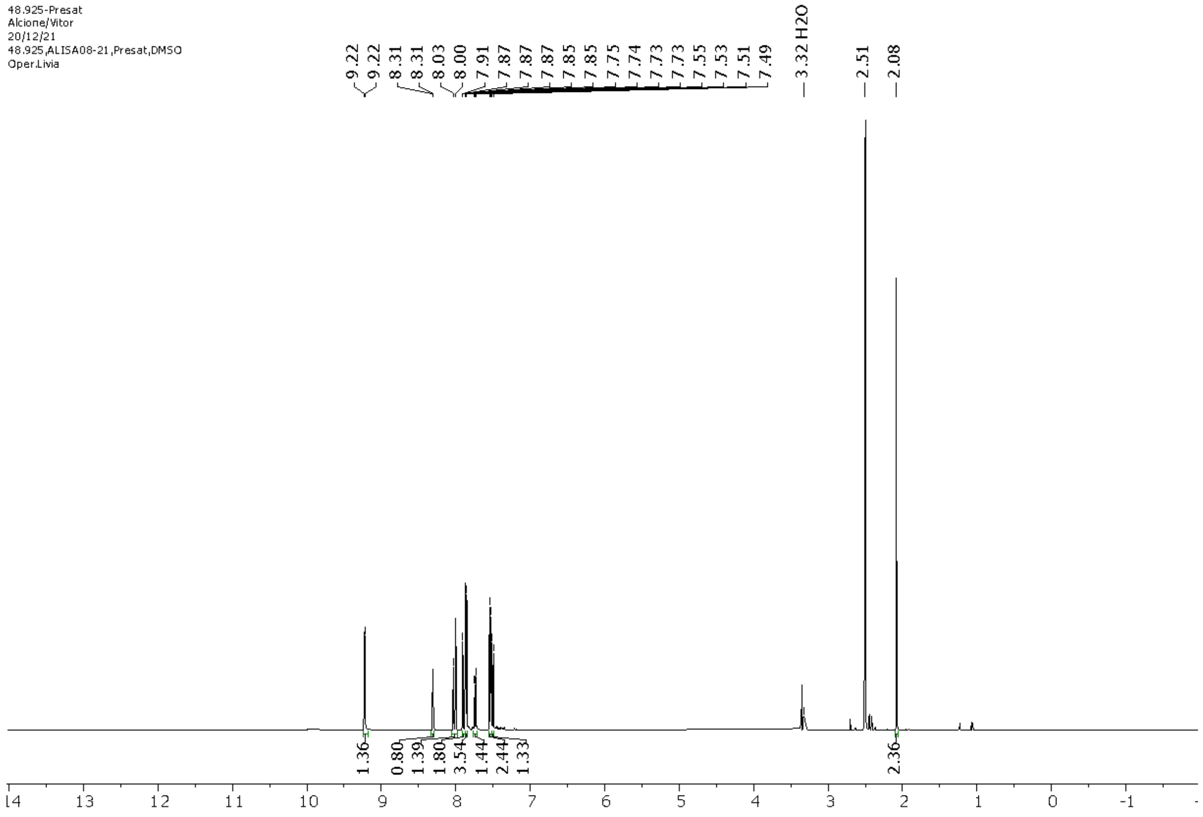

^{1}H-NMR SPECTRUM OF (*E*)-3-(4-CHLOROPHENYL)-1-(1-(7-CHLOROQUINOLIN-4-YL)-5-METHYL-1*H*-1,2,3-TRIAZOL-4-YL)PROP-2-EN-1-ONE **(4b)**.

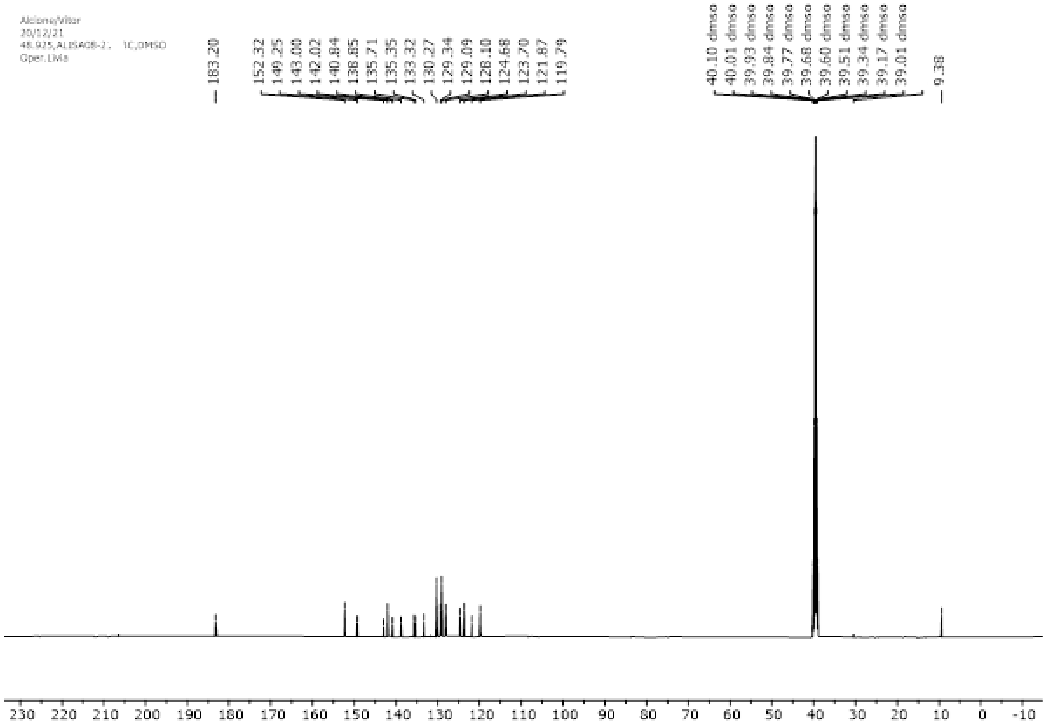

^{13}C-NMR SPECTRUM OF (*E*)-3-(4-CHLOROPHENYL)-1-(1-(7-CHLOROQUINOLIN-4-YL)-5-METHYL-1*H*-1,2,3-TRIAZOL-4-YL)PROP-2-EN-1-ONE **(4b)**.

+MS, 0.2-0.4min #9-25

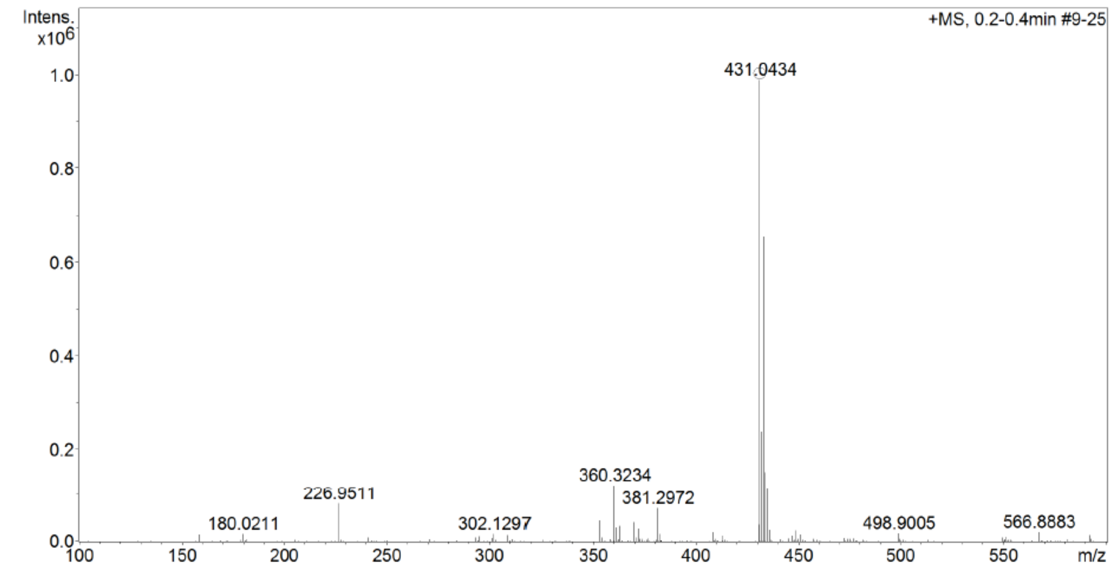

Meas. m/z	#	Ion Formula	m/z	err [ppm]	Mean err [ppm]	rdb	N-Rule	e⁻ Conf	mSigma	Std I	Std Mean m/z	Std I VarNorm	Std m/z Diff	Std Comb Dev
431.043448	1	C21H14Cl2N4NaO	431.043687	0.6	0.7	15.5	ok	even	4.7	5.6	n.a.	n.a.	n.a.	n.a.

HRMS OF (*E*)-3-(4-CHLOROPHENYL)-1-(1-(7-CHLOROQUINOLIN-4-YL)-5-METHYL-1*H*-1,2,3-TRIAZOL-4-YL)PROP-2-EN-1-ONE **(4b)**.

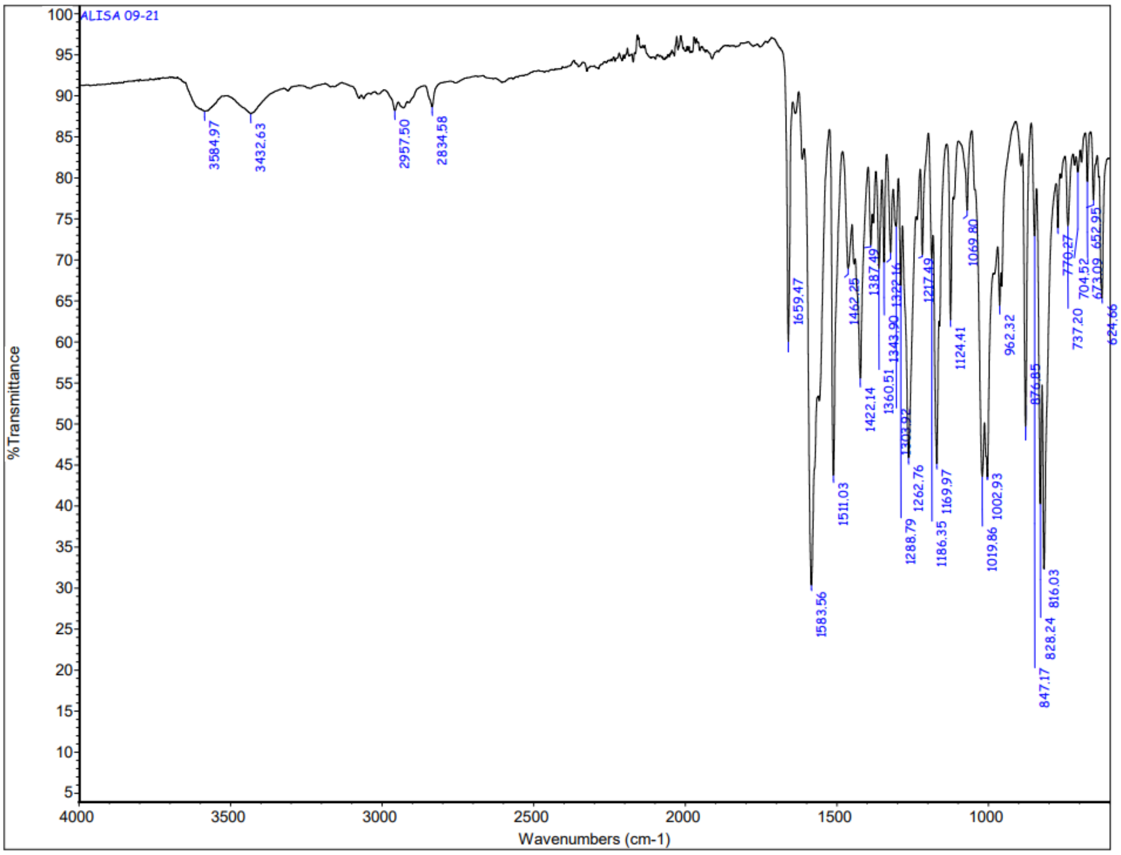

INFRARED SPECTRUM OF (*E*)-1-(1-(7-CHLOROQUINOLIN-4-YL)-5-METHYL-1*H*-1,2,3-TRIAZOL-4-YL)-3-(4-METHOXYPHENYL)PROP-2-EN-1-ONE (**4c**).

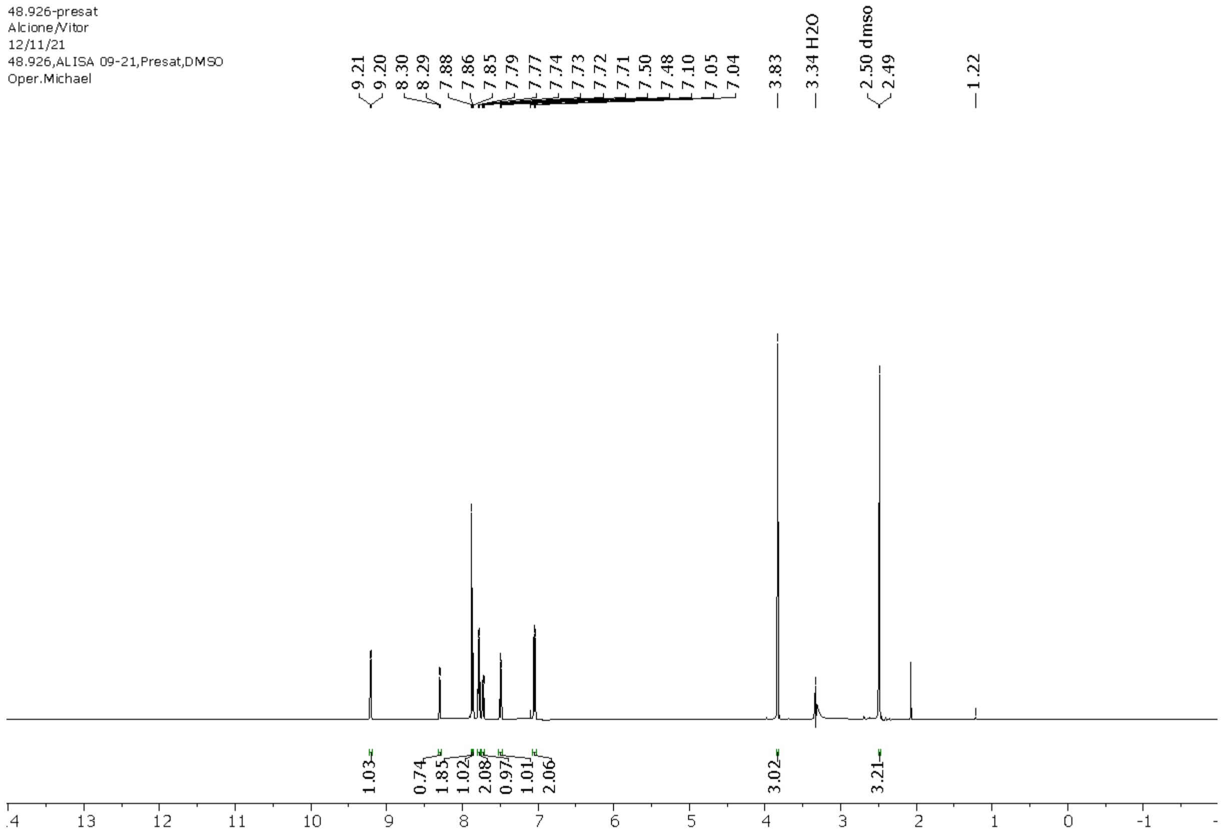

^{1}H-NMR SPECTRUM OF (*E*)-1-(1-(7-CHLOROQUINOLIN-4-YL)-5-METHYL-1*H*-1,2,3-TRIAZOL-4-YL)-3-(4-METHOXYPHENYL)PROP-2-EN-1-ONE (**4c**).

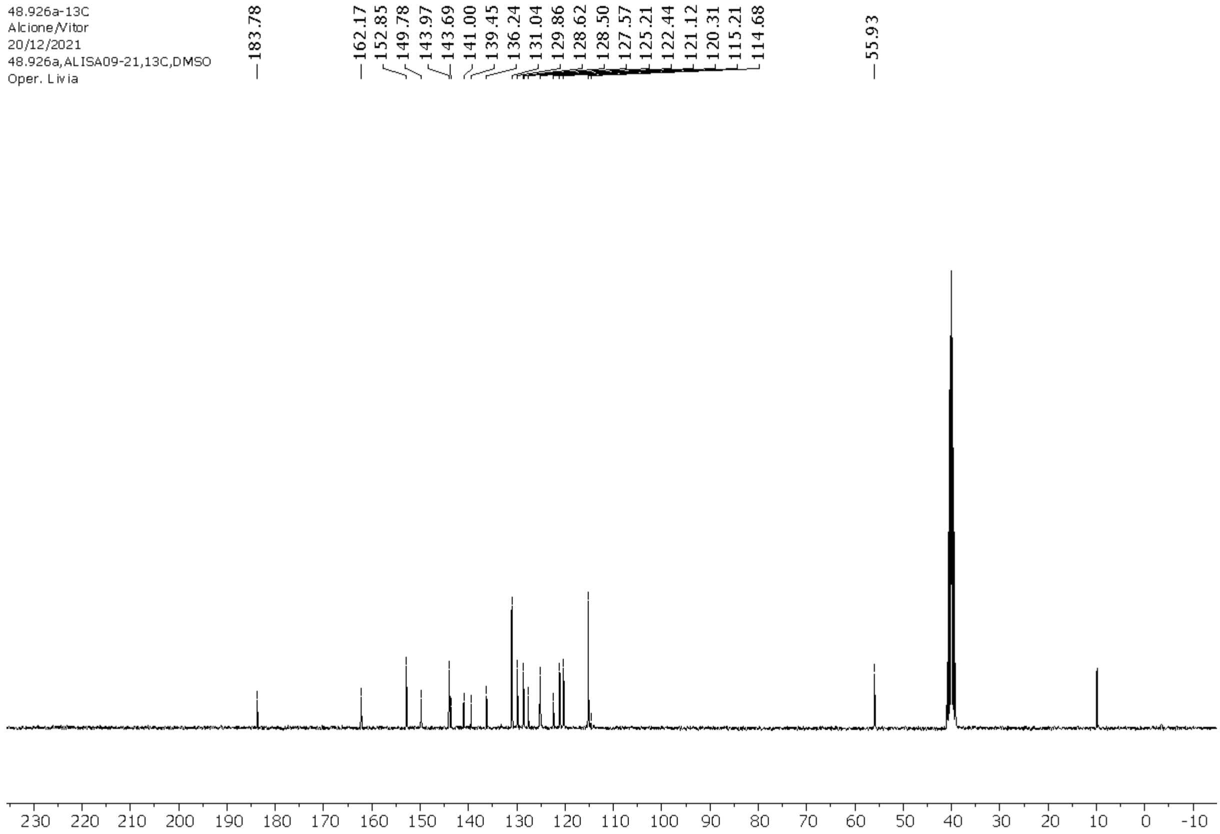

^{13}C-NMR SPECTRUM OF (*E*)-1-(1-(7-CHLOROQUINOLIN-4-YL)-5-METHYL-1*H*-1,2,3-TRIAZOL-4-YL)-3-(4-METHOXYPHENYL)PROP-2-EN-1-ONE (**4c**).

+MS, 0.1-0.4min #5-24

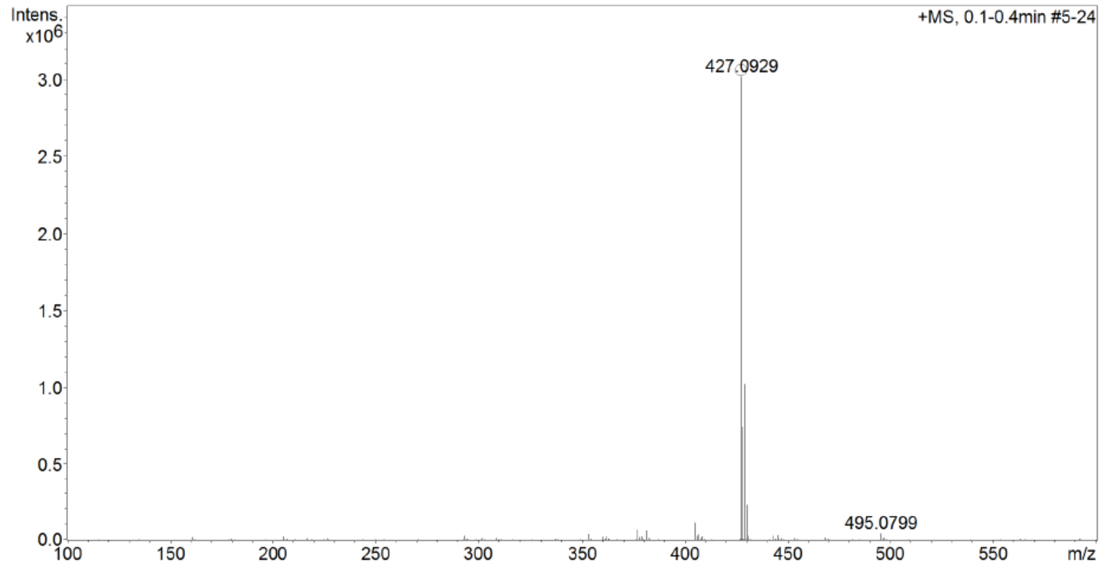

Meas. m/z	#	Ion Formula	m/z	err [ppm]	Mean err [ppm]	rdb	N-Rule	e⁻ Conf	mSigma	Std I	Std Mean m/z	Std I VarNorm	Std m/z Diff	Std Comb Dev
427.092915	1	C22H17ClN4NaO2	427.093224	0.7	0.8	15.5	ok	even	8.6	13.5	n.a.	n.a.	n.a.	n.a.

HRMS OF (*E*)-1-(1-(7-CHLOROQUINOLIN-4-YL)-5-METHYL-1*H*-1,2,3-TRIAZOL-4-YL)-3-(4-METHOXYPHENYL)PROP-2-EN-1-ONE (**4c**).

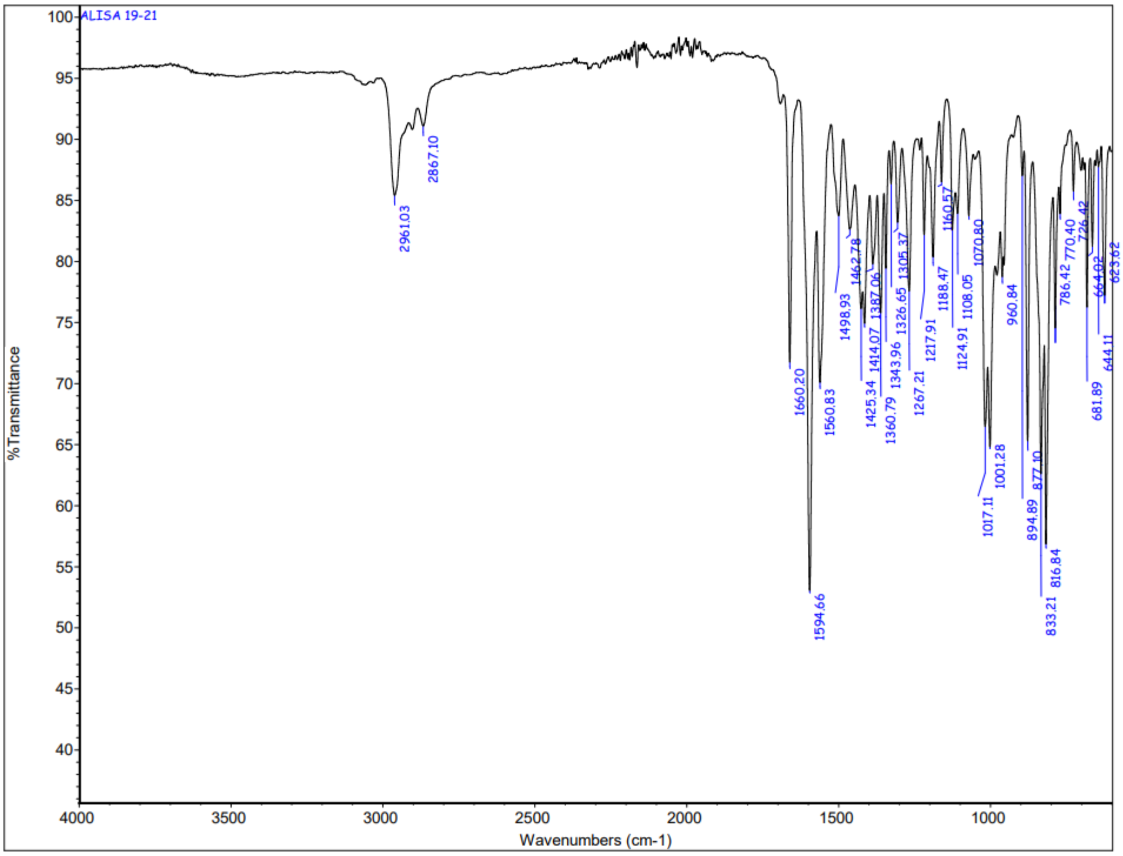

INFRARED SPECTRUM OF (*E*)-3-(4-(TERT-BUTYL)PHENYL)-1-(1-(7-CHLOROQUINOLIN-4-YL)-5-METHYL-1*H*-1,2,3-TRIAZOL-4-YL)PROP-2-EN-1-ONE (**4d**).

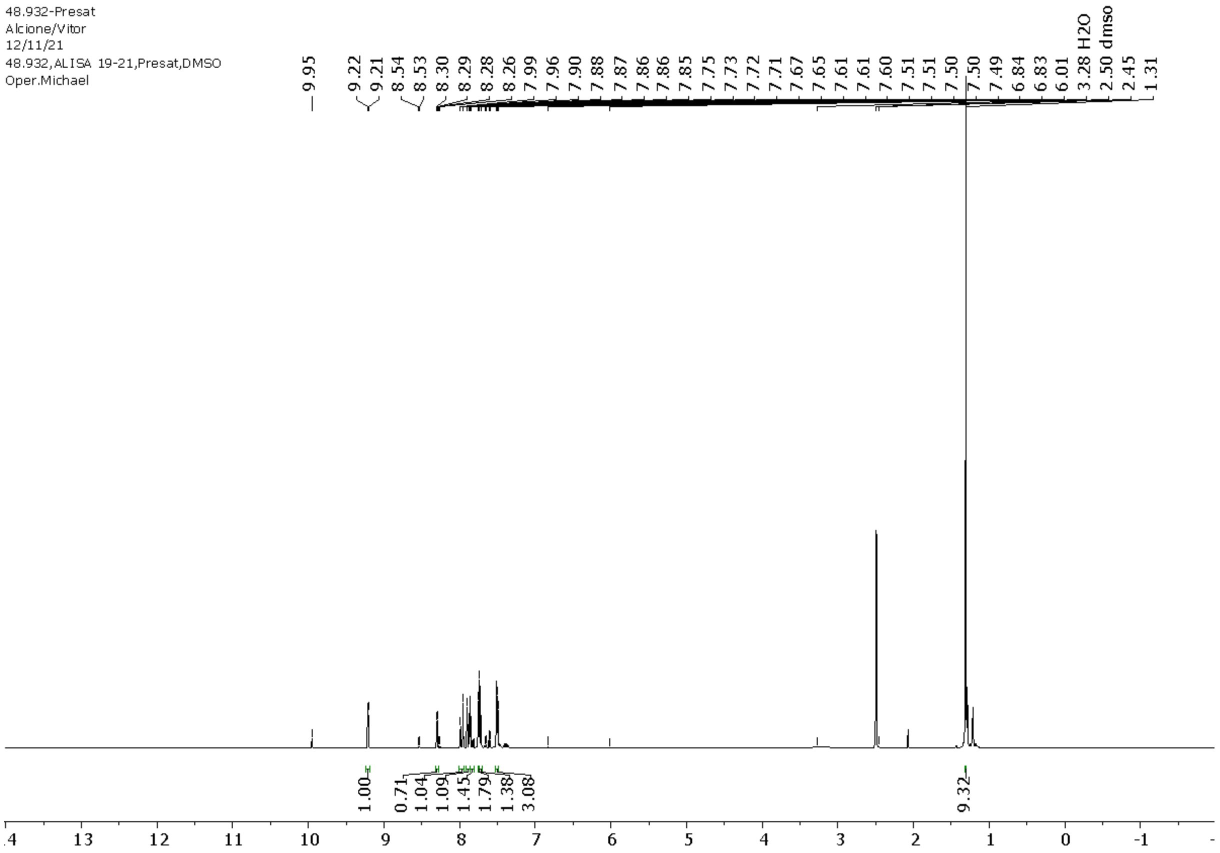

^{1}H-NMR SPECTRUM OF (*E*)-3-(4-(TERT-BUTYL)PHENYL)-1-(1-(7-CHLOROQUINOLIN-4-YL)-5-METHYL-1*H*-1,2,3-TRIAZOL-4-YL)PROP-2-EN-1-ONE (**4d**).

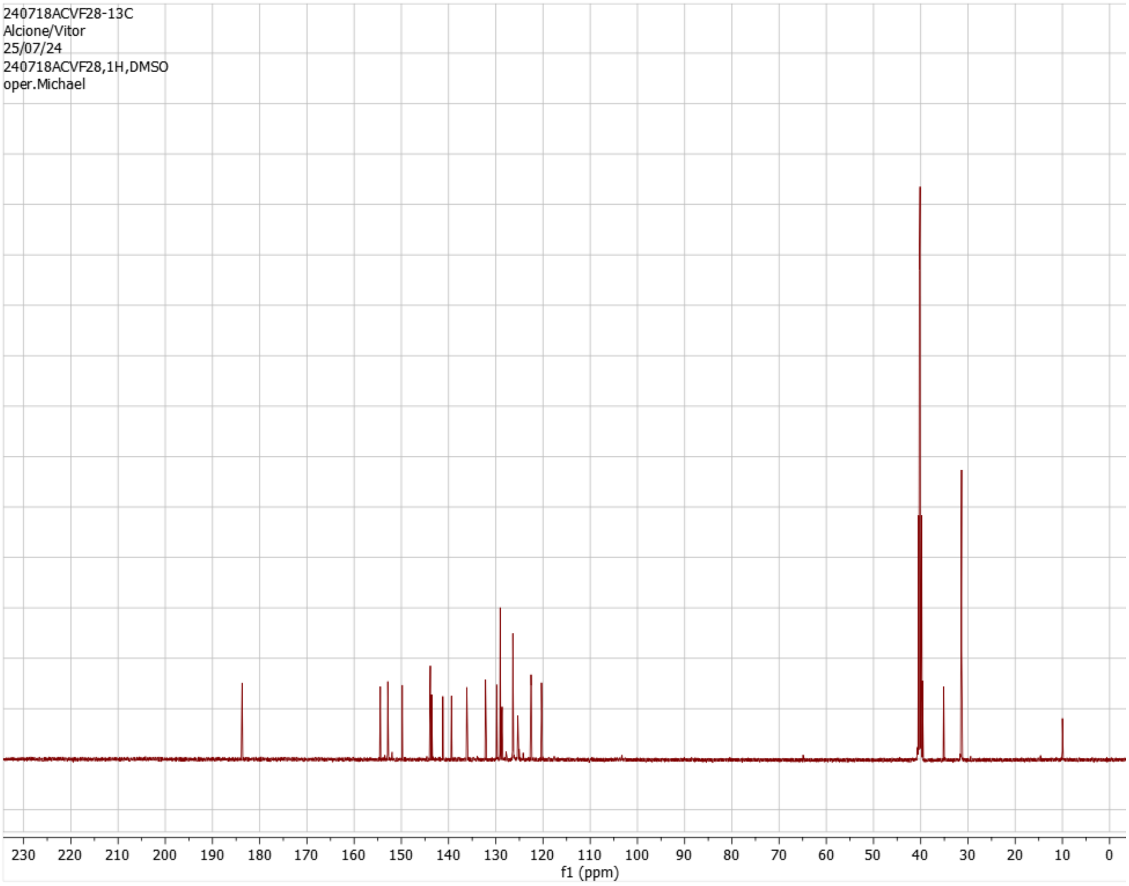

^{13}C-NMR SPECTRUM OF (*E*)-3-(4-(TERT-BUTYL)PHENYL)-1-(1-(7-CHLOROQUINOLIN-4-YL)-5-METHYL-1*H*-1,2,3-TRIAZOL-4-YL)PROP-2-EN-1-ONE (**4d**).

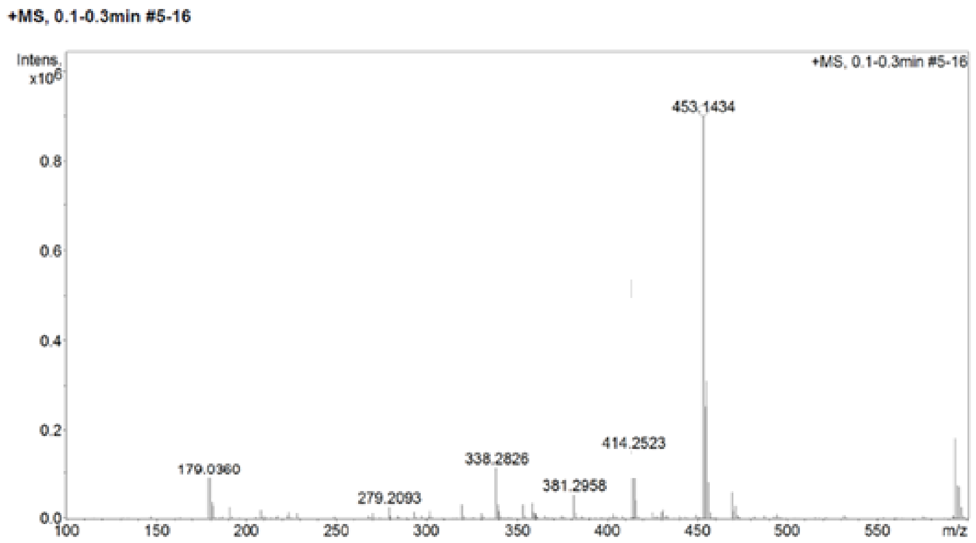

Meas. m/z	#	Ion Formula	m/z	err [ppm]	Mean err [ppm]	rdb	N-Rule	e⁻ Conf	mSigma	Std I	Std Mean m/z	Std I VarNorm	Std m/z Diff	Std Comb Dev
453.143410	1	C25H23ClN4NaO	453.145260	4.1	5.7	15.5	ok	even	8.5	11.4	n.a.	n.a.	n.a.	n.a.

HRMS OF (*E*)-3-(4-(TERT-BUTYL)PHENYL)-1-(1-(7-CHLOROQUINOLIN-4-YL)-5-METHYL-1*H*-1,2,3-TRIAZOL-4-YL)PROP-2-EN-1-ONE (**4d**).

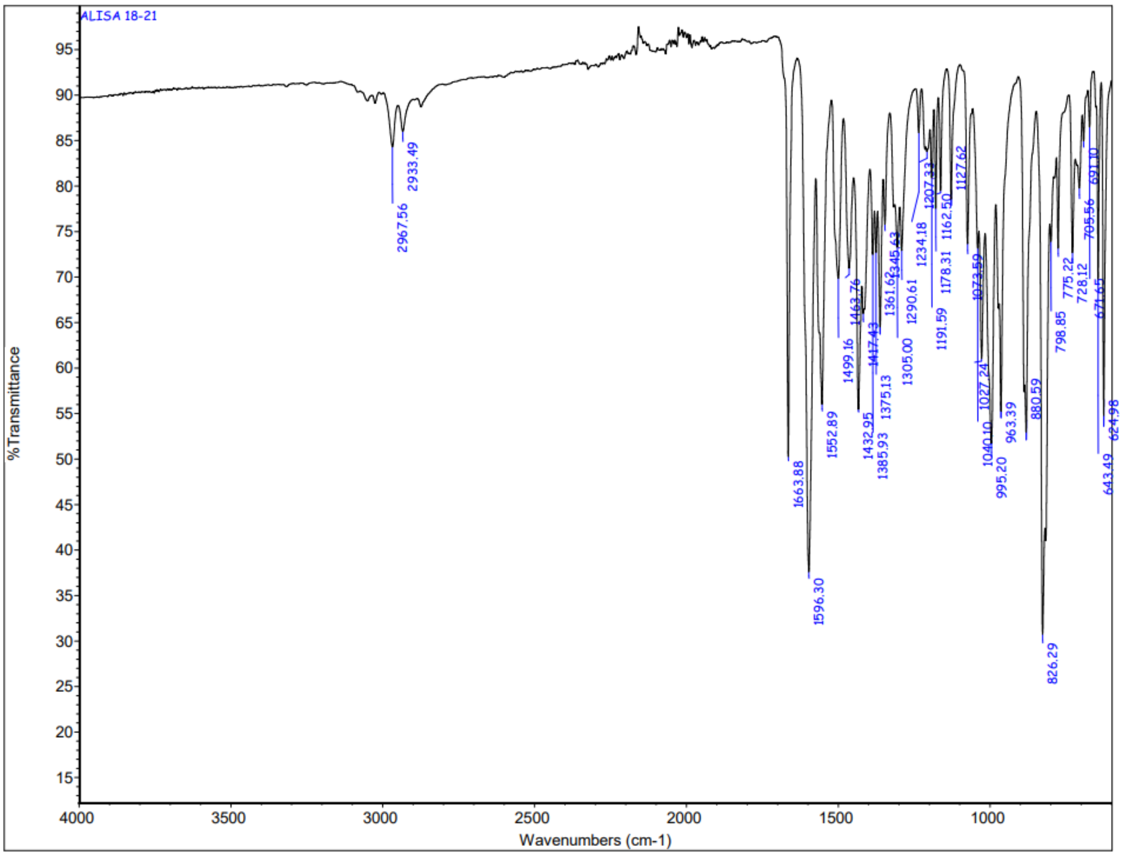

INFRARED SPECTRUM OF (*E*)-1-(1-(7-CHLOROQUINOLIN-4-YL)-5-METHYL-1*H*-1,2,3-TRIAZOL-4-YL)-3-(4-ETHYLPHENYL)PROP-2-EN-1-ONE **(4e)**.

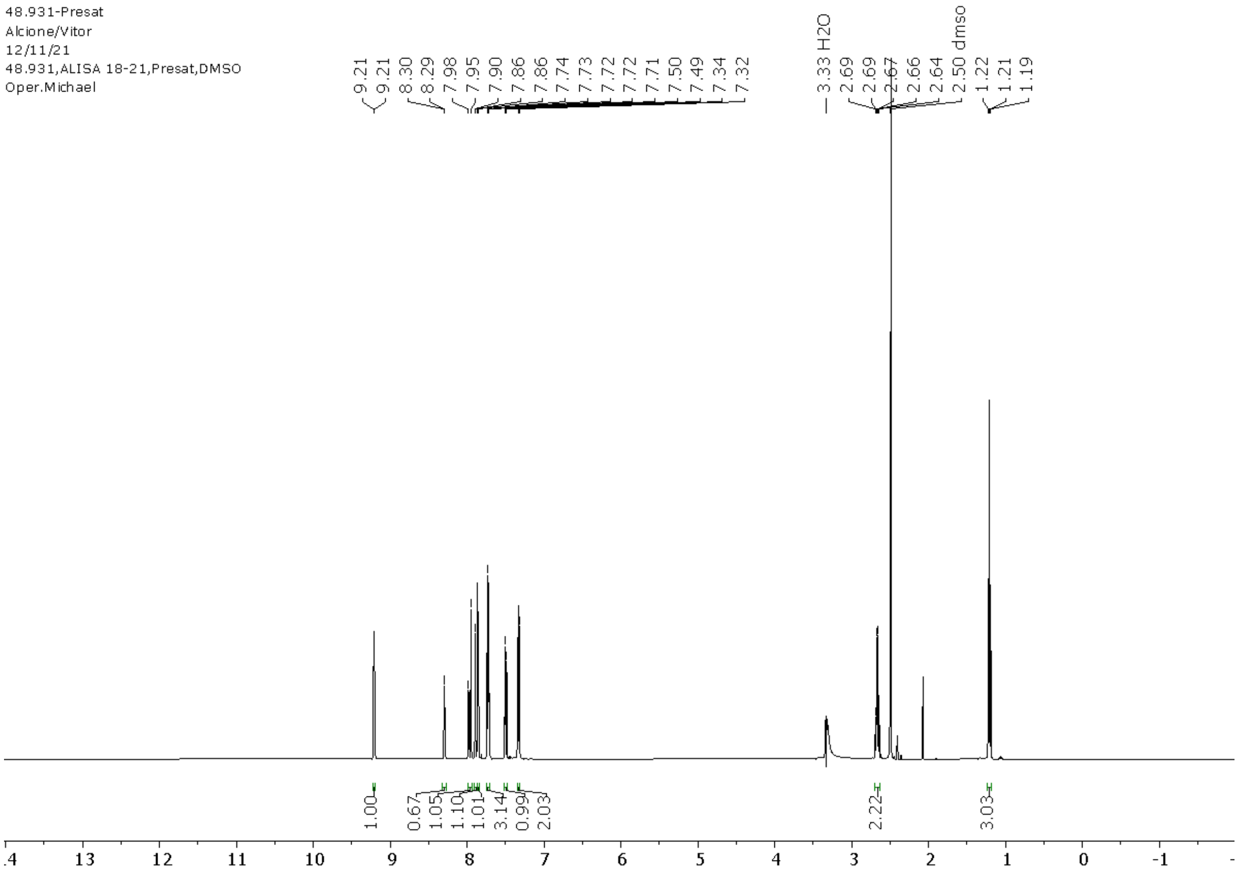

^{1}H-NMR SPECTRUM OF (*E*)-1-(1-(7-CHLOROQUINOLIN-4-YL)-5-METHYL-1*H*-1,2,3-TRIAZOL-4-YL)-3-(4-ETHYLPHENYL)PROP-2-EN-1-ONE **(4e)**.

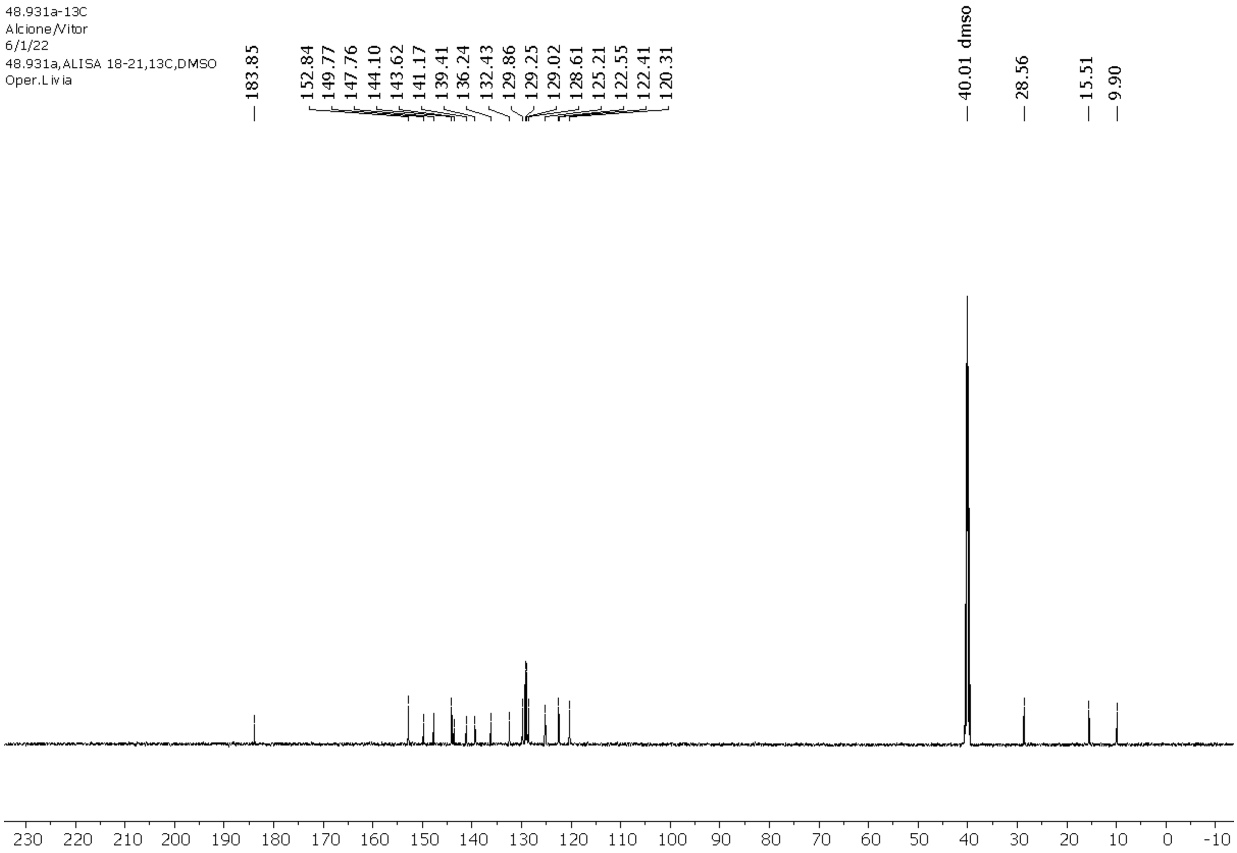

^{13}C-NMR SPECTRUM OF (*E*)-1-(1-(7-CHLOROQUINOLIN-4-YL)-5-METHYL-1*H*-1,2,3-TRIAZOL-4-YL)-3-(4-ETHYLPHENYL)PROP-2-EN-1-ONE **(4e)**.

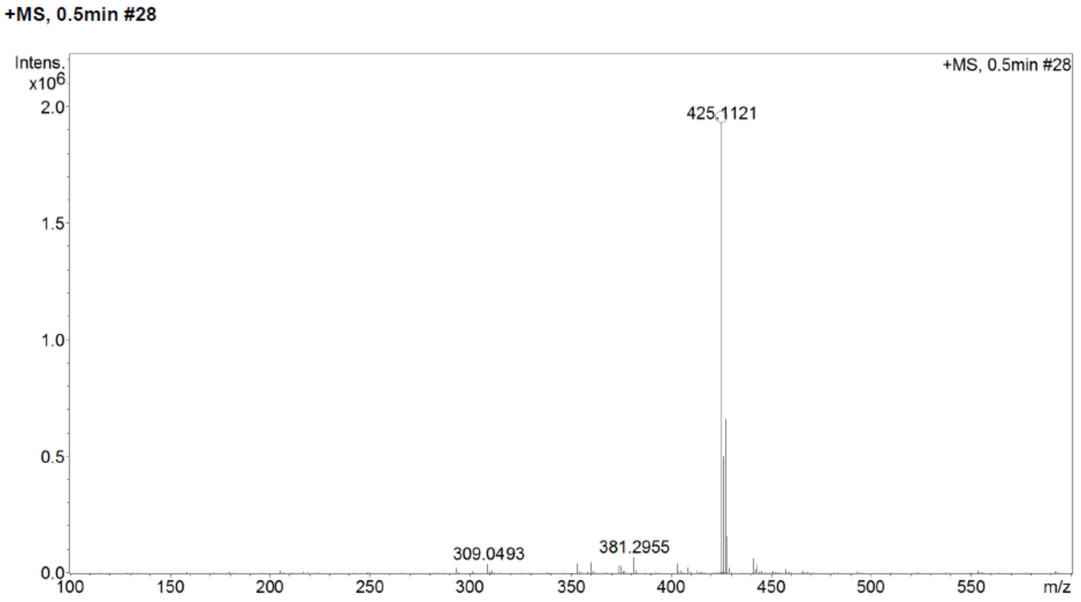

Meas. m/z	#	Ion Formula	m/z	err [ppm]	Mean err [ppm]	rdb	N-Rule	e⁻ Conf	mSigma	Std I	Std Mean m/z	Std I VarNorm	Std m/z Diff	Std Comb Dev
425.112112	1	C23H19ClN4NaO	425.113960	4.3	5.0	15.5	ok	even	6.4	9.7	n.a.	n.a.	n.a.	n.a.

HRMS OF (*E*)-1-(1-(7-CHLOROQUINOLIN-4-YL)-5-METHYL-1*H*-1,2,3-TRIAZOL-4-YL)-3-(4-ETHYLPHENYL)PROP-2-EN-1-ONE **(4e)**.

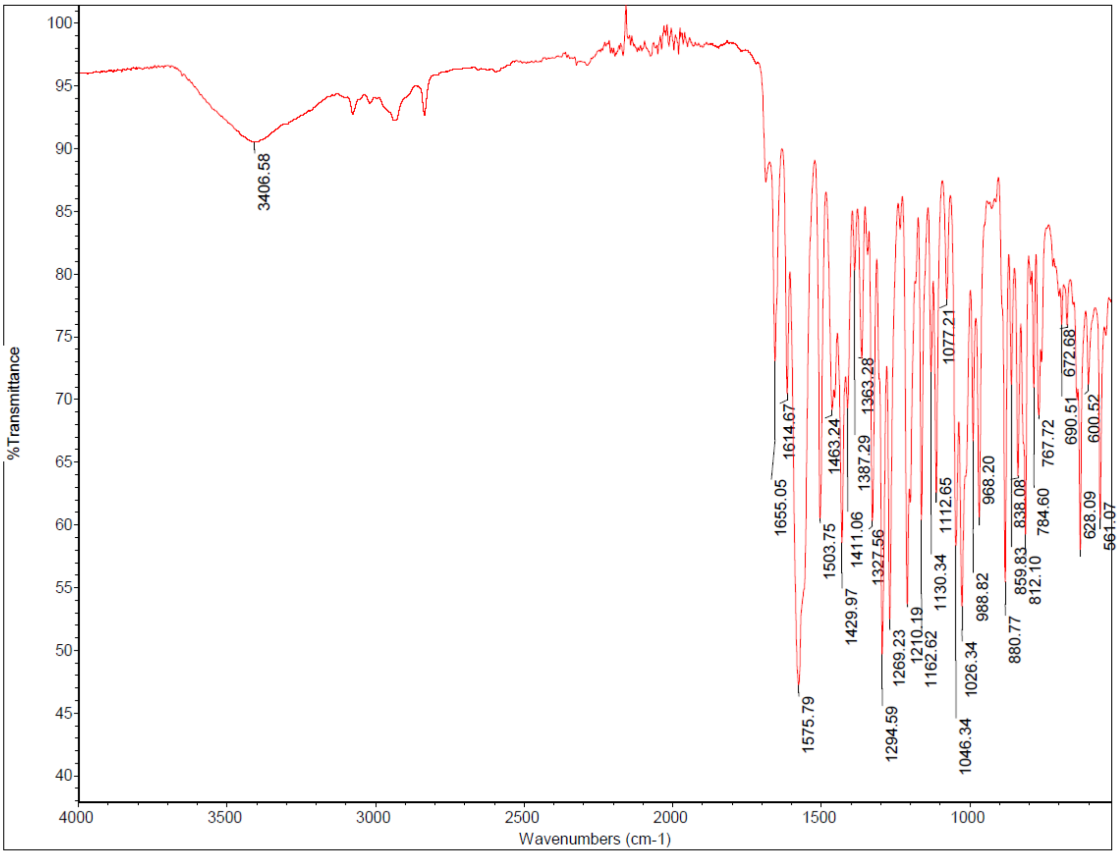

INFRARED SPECTRUM OF (*E*)-1-(1-(7-CHLOROQUINOLIN-4-YL)-5-METHYL-1*H*-1,2,3-TRIAZOL-4-YL)-3-(2,4-DIMETHOXYPHENYL)PROP-2-EN-1-ONE **(4f)**.

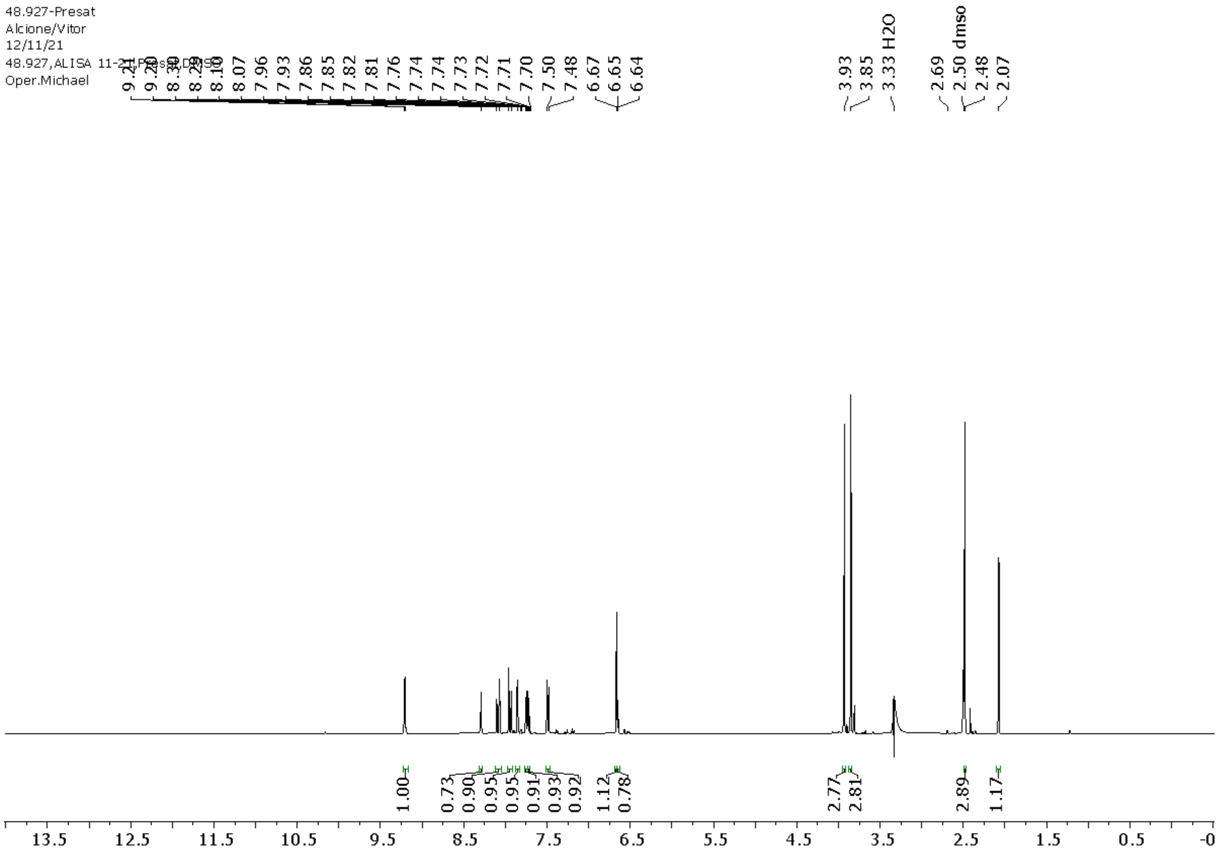

^{1}H-NMR SPECTRUM OF (*E*)-1-(1-(7-CHLOROQUINOLIN-4-YL)-5-METHYL-1*H*-1,2,3-TRIAZOL-4-YL)-3-(2,4-DIMETHOXYPHENYL)PROP-2-EN-1-ONE **(4f)**.

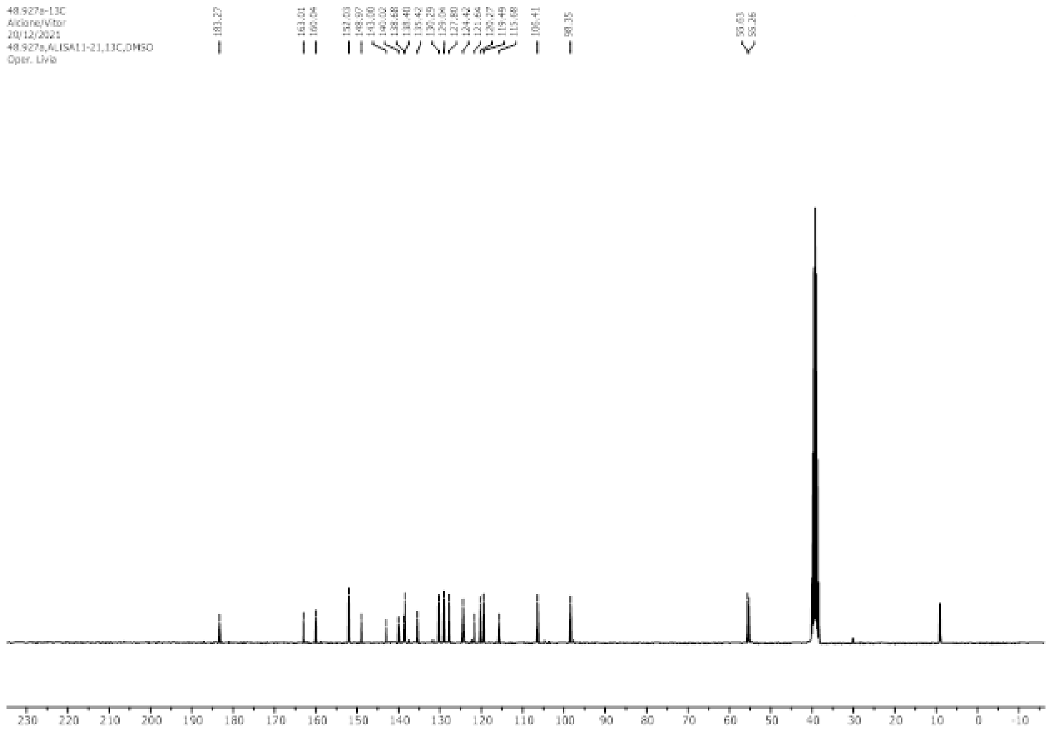

^{13}C-NMR SPECTRUM OF (*E*)-1-(1-(7-CHLOROQUINOLIN-4-YL)-5-METHYL-1*H*-1,2,3-TRIAZOL-4-YL)-3-(2,4-DIMETHOXYPHENYL)PROP-2-EN-1-ONE **(4f)**.

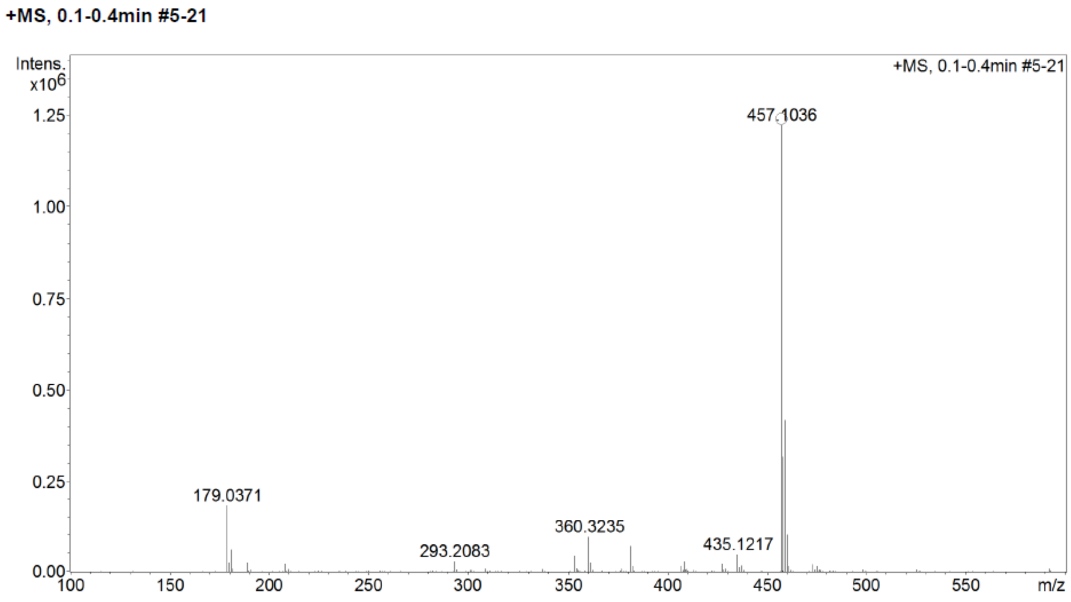

Meas. m/z	#	Ion Formula	m/z	err [ppm]	Mean err [ppm]	rdb	N-Rule	e⁻ Conf	mSigma	Std I	Std Mean m/z	Std I VarNorm	Std m/z Diff	Std Comb Dev
457.103603	1	C23H19ClN4NaO3	457.103789	0.4	0.4	15.5	ok	even	8.6	12.4	n.a.	n.a.	n.a.	n.a.

HRMS OF (*E*)-1-(1-(7-CHLOROQUINOLIN-4-YL)-5-METHYL-1*H*-1,2,3-TRIAZOL-4-YL)-3-(2,4-DIMETHOXYPHENYL)PROP-2-EN-1-ONE **(4f)**.

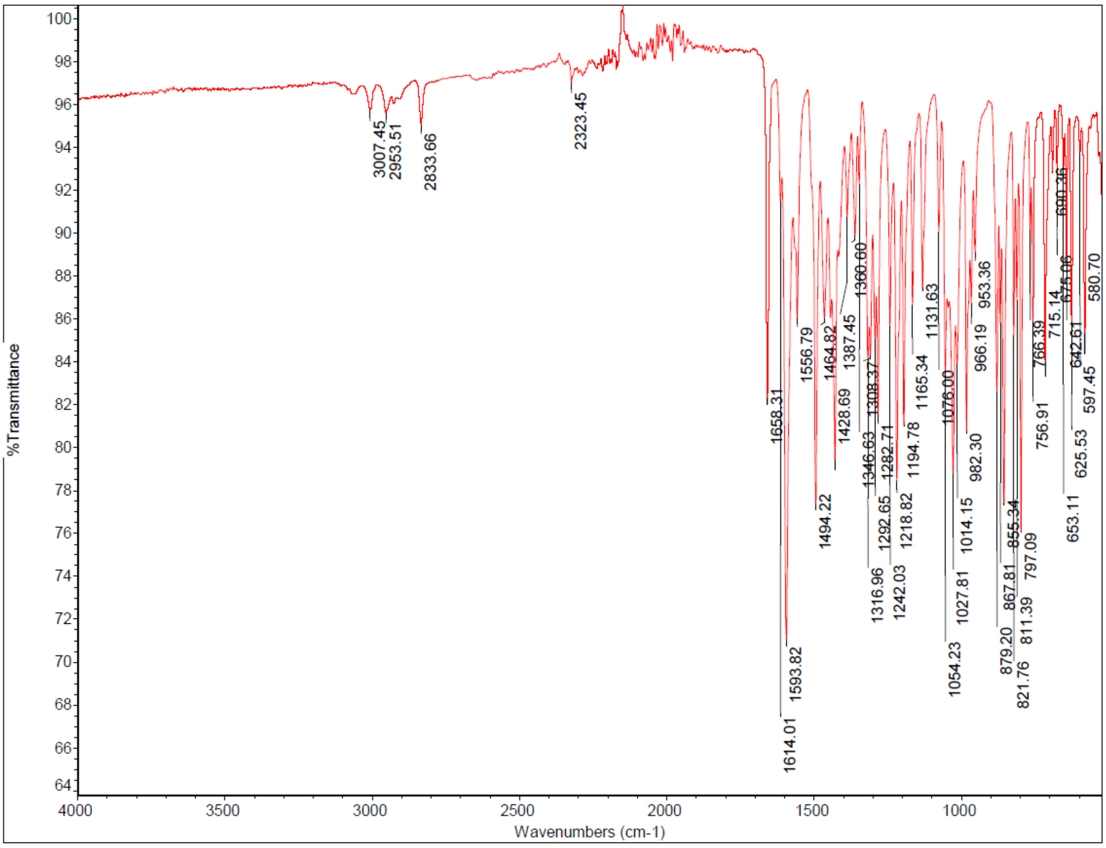

INFRARED SPECTRUM OF **(***E***)**-1-(1-(7-CHLOROQUINOLIN-4-YL)-5-METHYL-1*H*-1,2,3-TRIAZOL-4-YL)-3-(3,4-DIMETHOXYPHENYL)PROP-2-EN-1-ONE **(4g)**.

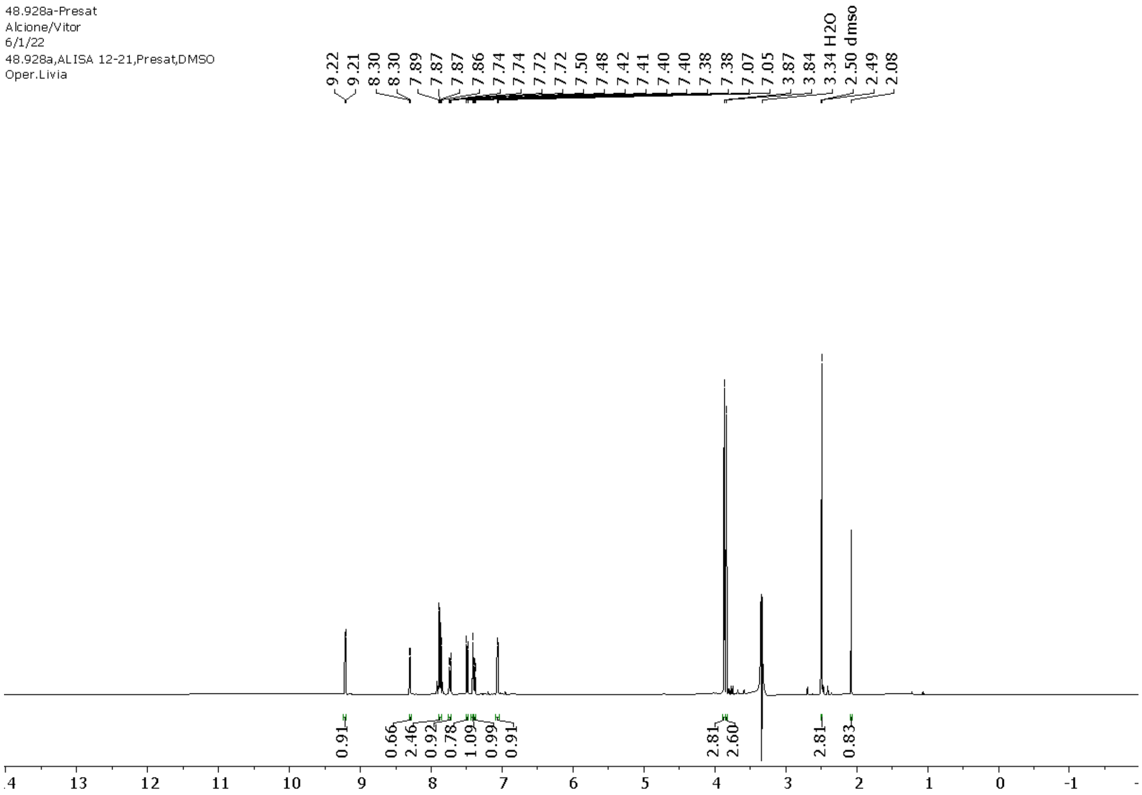

^{1}H-NMR SPECTRUM OF (*E*)-1-(1-(7-CHLOROQUINOLIN-4-YL)-5-METHYL-1*H*-1,2,3-TRIAZOL-4-YL)-3-(3,4-DIMETHOXYPHENYL)PROP-2-EN-1-ONE **(4g)**.

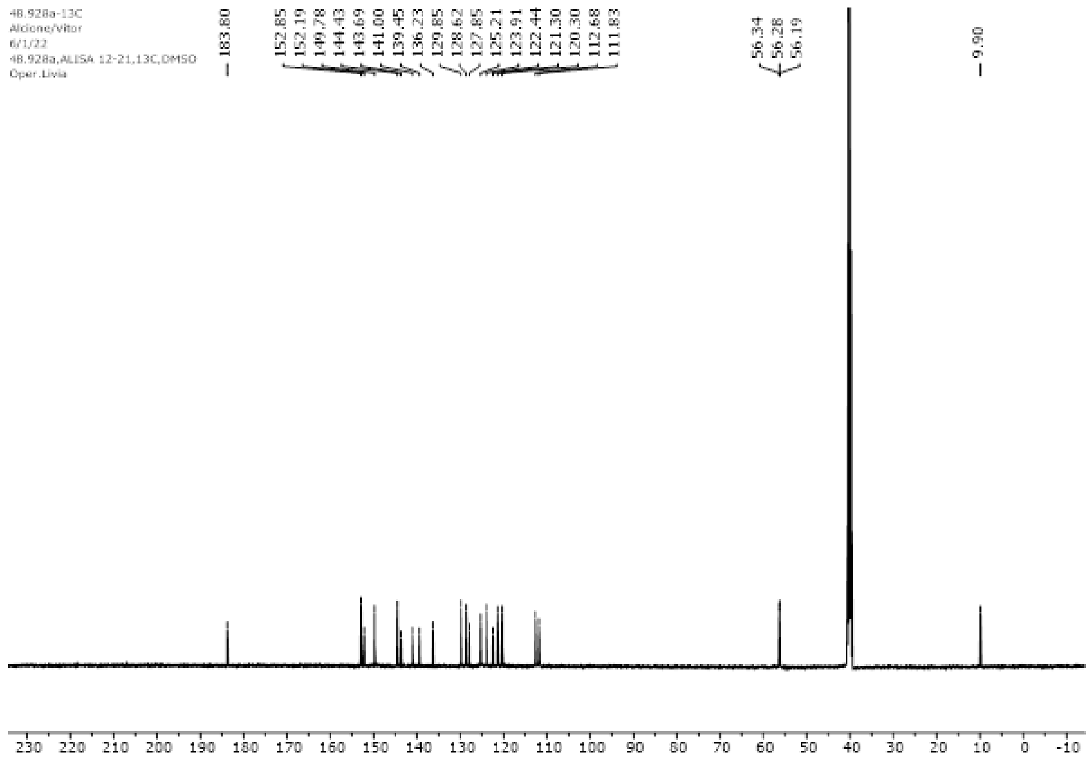

^{13}C-NMR SPECTRUM OF **(***E***)**-1-(1-(7-CHLOROQUINOLIN-4-YL)-5-METHYL-1*H*-1,2,3-TRIAZOL-4-YL)-3-(3,4-DIMETHOXYPHENYL)PROP-2-EN-1-ONE **(4g)**.

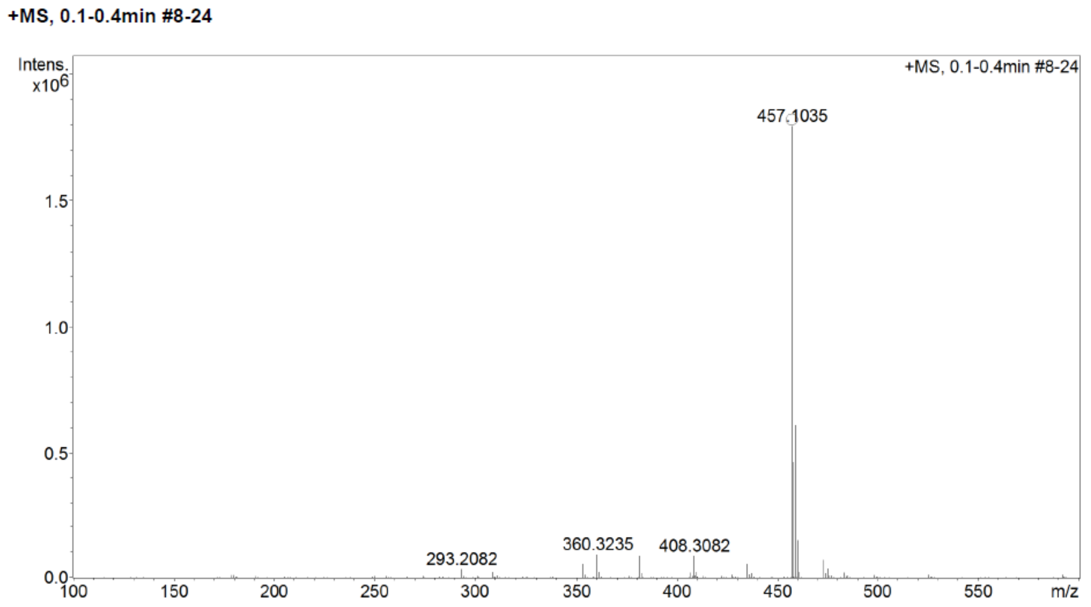

Meas. m/z	#	Ion Formula	m/z	err [ppm]	Mean err [ppm]	rdb	N-Rule	e⁻ Conf	mSigma	Std I	Std Mean m/z	Std I VarNorm	Std m/z Diff	Std Comb Dev
457.103505	1	C23H19ClN4NaO3	457.103789	0.6	0.8	15.5	ok	even	9.5	13.8	n.a.	n.a.	n.a.	n.a.

HRMS OF **(***E***)**-1-(1-(7-CHLOROQUINOLIN-4-YL)-5-METHYL-1*H*-1,2,3-TRIAZOL-4-YL)-3-(3,4-DIMETHOXYPHENYL)PROP-2-EN-1-ONE **(4g)**.

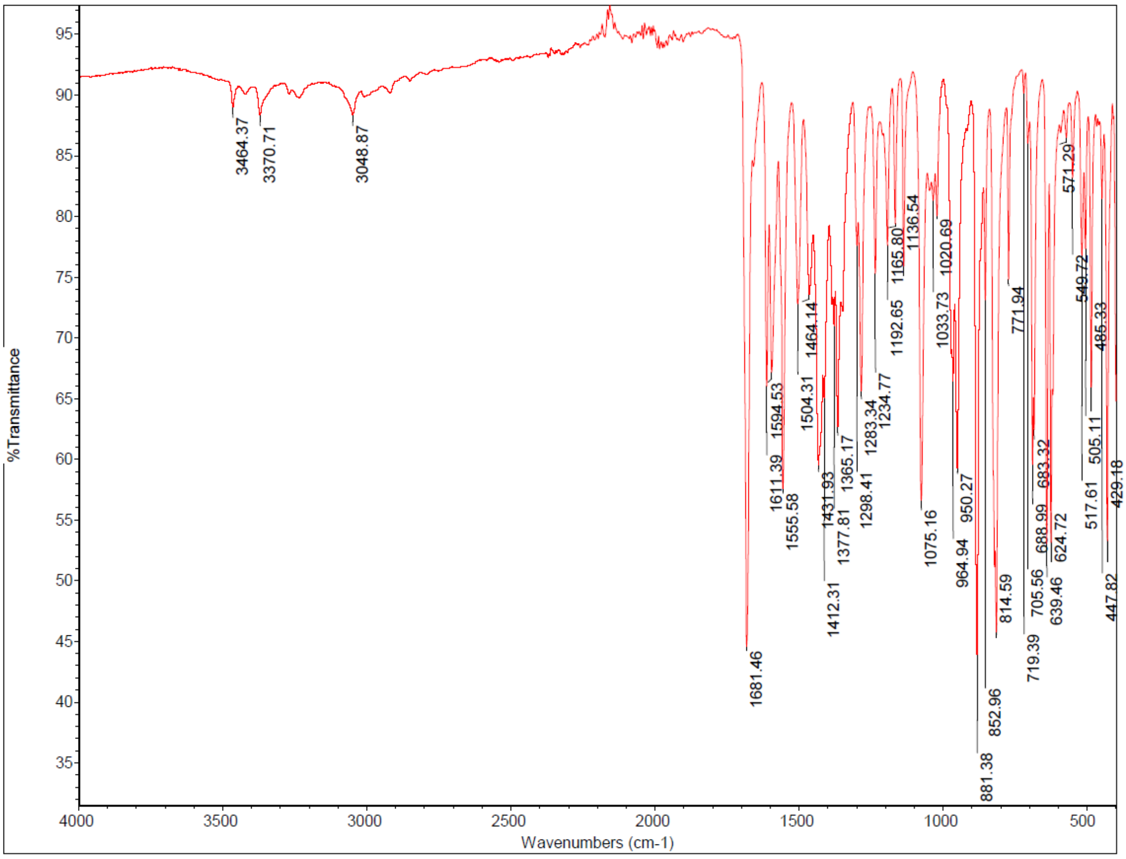

INFRARED SPECTRUM OF (*E*)-1-(1-(7-CHLOROQUINOLIN-4-YL)-5-METHYL-1*H*-1,2,3-TRIAZOL-4-YL)-3-(2,5-DIMETHOXYPHENYL)PROP-2-EN-1-ONE **(4h)**.

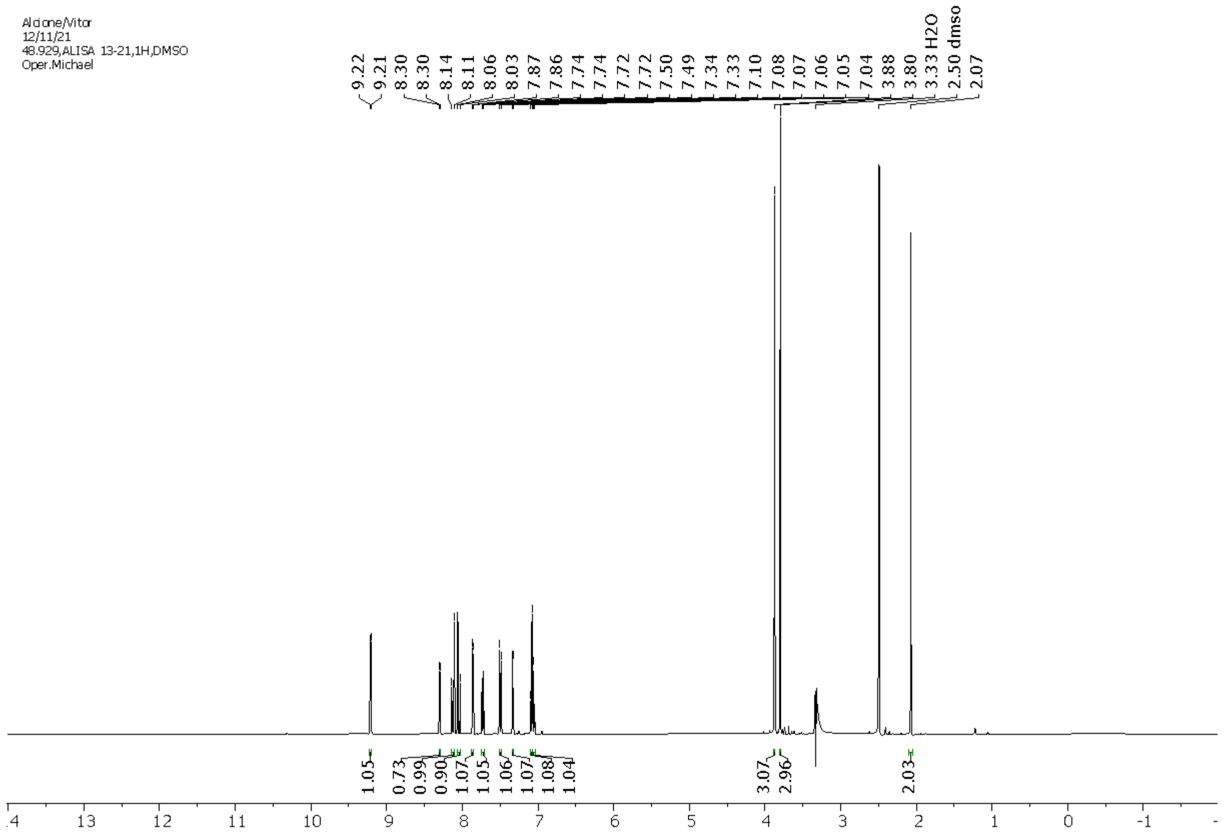

^{1}H-NMR SPECTRUM OF (*E*)-1-(1-(7-CHLOROQUINOLIN-4-YL)-5-METHYL-1*H*-1,2,3-TRIAZOL-4-YL)-3-(2,5-DIMETHOXYPHENYL)PROP-2-EN-1-ONE **(4h)**.

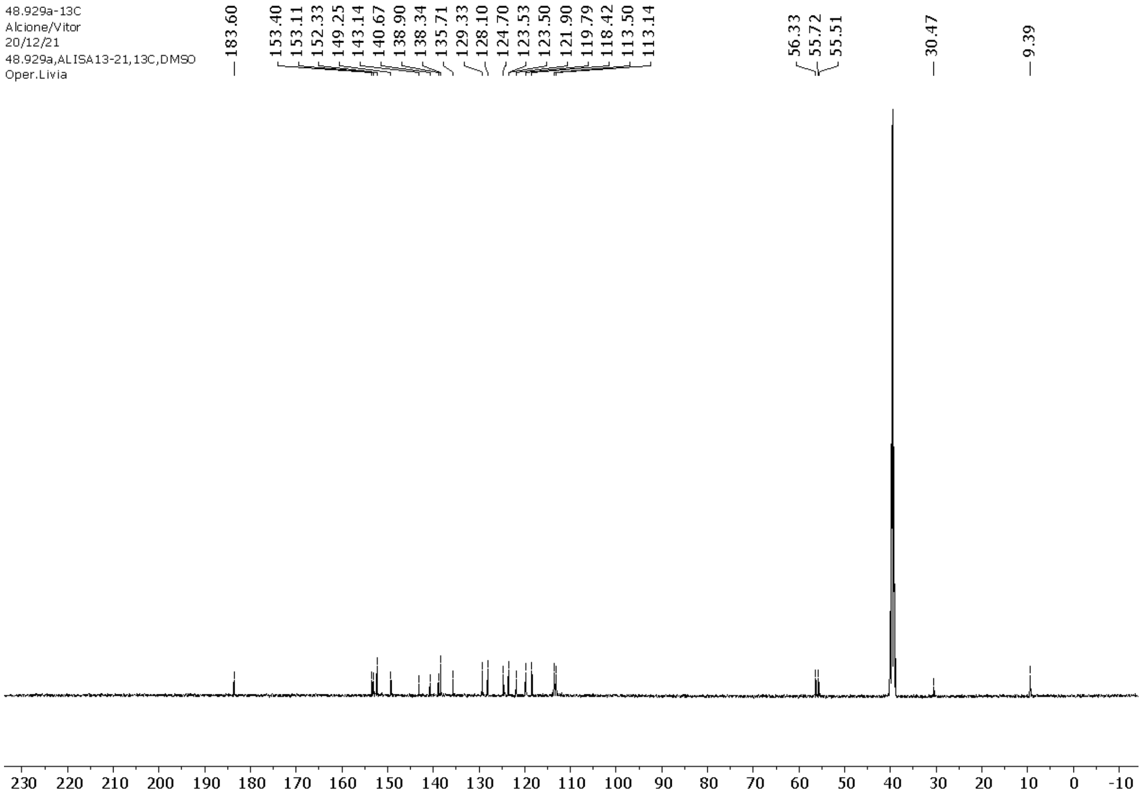

^{13}C-NMR SPECTRUM OF **(**E**)**-1-(1-(7-CHLOROQUINOLIN-4-YL)-5-METHYL-1H-1,2,3-TRIAZOL-4-YL)-3-(2,5-DIMETHOXYPHENYL)PROP-2-EN-1-ONE **(4h)**.

+MS, 0.4-0.7min #23-38

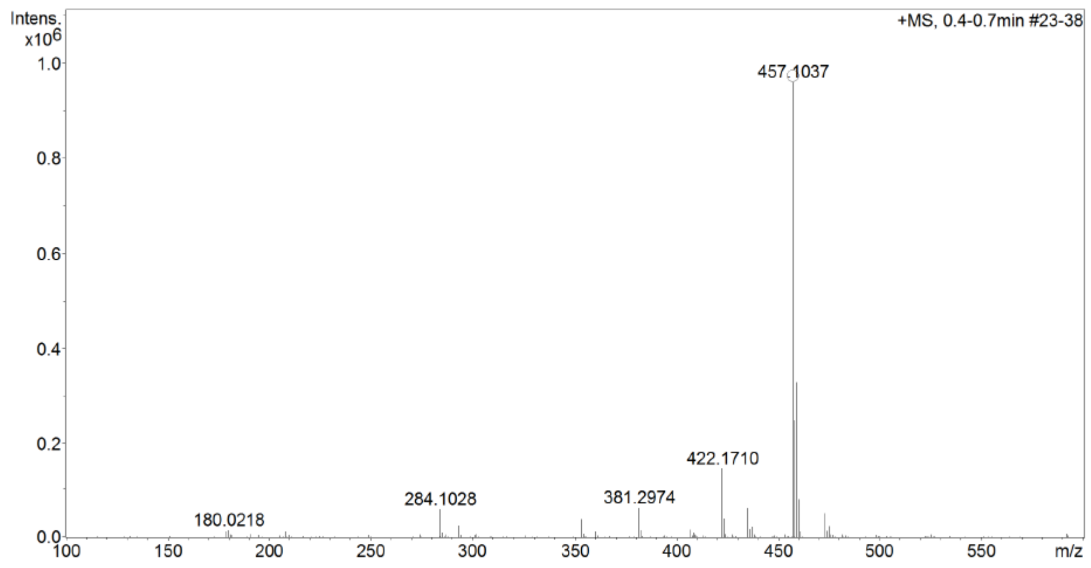

Meas. m/z	#	Ion Formula	m/z	err [ppm]	Mean err [ppm]	rdb	N-Rule	e⁻ Conf	mSigma	Std I	Std Mean m/z	Std I VarNorm	Std m/z Diff	Std Comb Dev
457.103672	1	C23H19ClN4NaO3	457.103789	0.3	0.3	15.5	ok	even	8.1	11.6	n.a.	n.a.	n.a.	n.a.

HRMS OF **(**_E_)-1-(1-(7-CHLOROQUINOLIN-4-YL)-5-METHYL-1_H_-1,2,3-TRIAZOL-4-YL)-3-(2,5-DIMETHOXYPHENYL)PROP-2-EN-1-ONE **(4h)**.

SMILES

3:

ClC1=CC=C2C(N=CC=C2N3C(C)=C(C(C)=O)N=N3)=C1

4a:

ClC1=CC=C2C(N=CC=C2N3C(C)=C(C(/C=C/C4=CC=C(F)C=C4)=O)N=N3)=C1

4b:

CC1=C(C(/C=C/C2=CC=C(Cl)C=C2)=O)N=NN1C3=CC=NC4=CC(Cl)=CC=C43

4c:
ClC1=CC=C2C(N=CC=C2N3C(C)=C(C(/C=C/C4=CC=C(OC)C=C4)=O)N=N3)=C1

4d:

ClC1=CC=C2C(N=CC=C2N3C(C)=C(C(/C=C/C4=CC=C(C(C)(C)C)C=C4)=O)N=N3
)=C1

4e:

ClC1=CC=C2C(N=CC=C2N3C(C)=C(C(/C=C/C4=CC=C(CC)C=C4)=O)N=N3)=C1

4f:

ClC1=CC=C2C(N=CC=C2N3C(C)=C(C(/C=C/C4=CC=C(OC)C=C4OC)=O)N=N3)=
C1

4g:

ClC1=CC=C2C(N=CC=C2N3C(C)=C(C(/C=C/C4=CC=C(OC)C(OC)=C4)=O)N=N3)
=C1

4h:

ClC1=CC=C2C(N=CC=C2N3C(C)=C(C(/C=C/C4=CC(OC)=CC=C4OC)=O)N=N3)=
C1

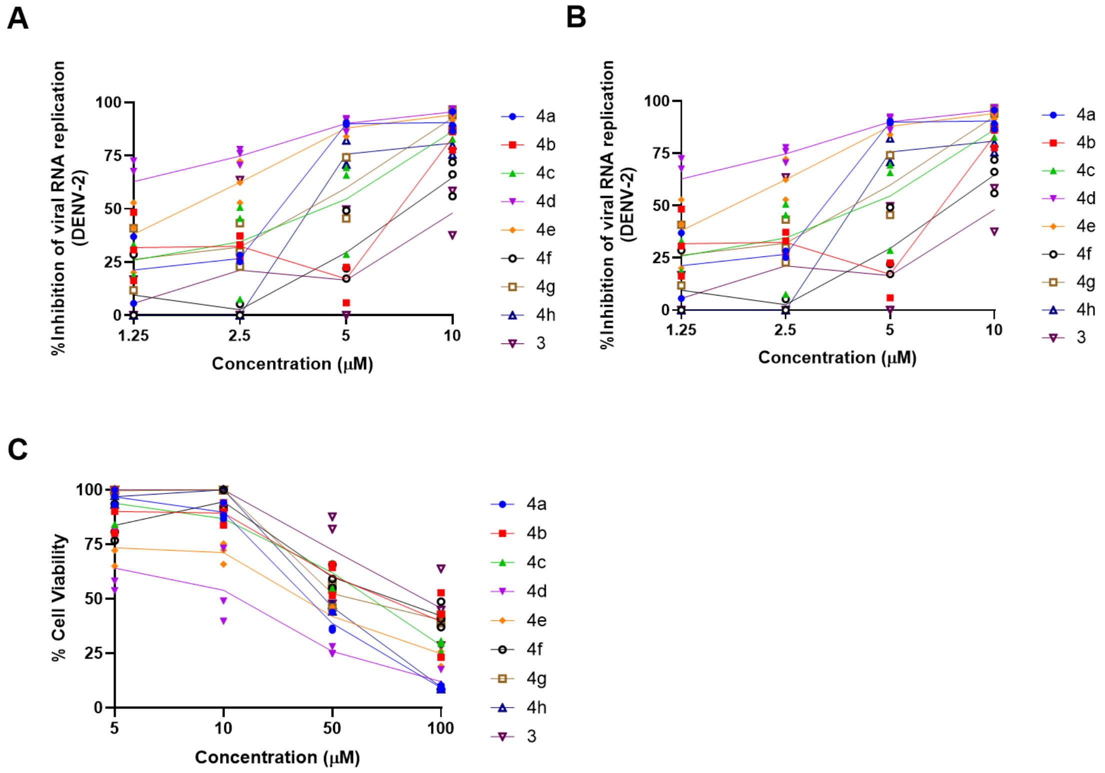

Fig S1. Dose-response curves and viability in Huh-7 cells. Huh-7 cells were infected with **(A)** CHIKV or **(B)** DENV-2 and treated with concentrations ranging from 1.25 to 10 μM of the molecules**.** Cell viability was also evaluated **(C)** after adding different concentrations of the compounds.

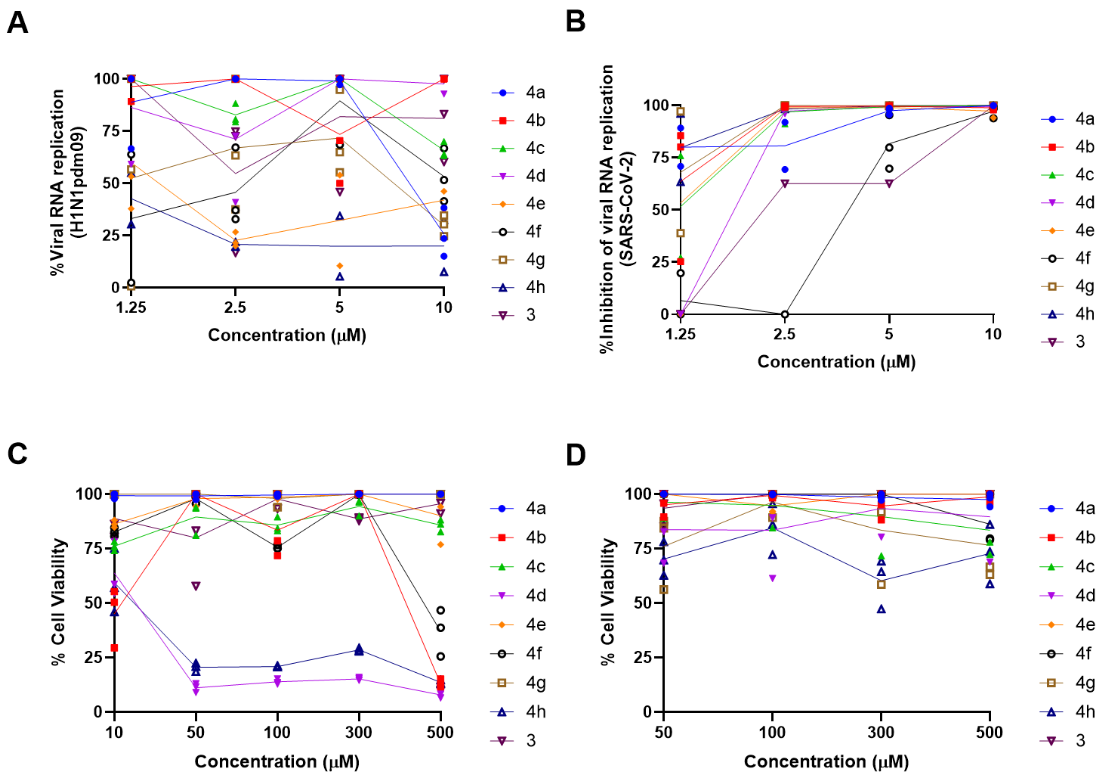

Fig S2. Dose-response curves and viability in MDCK and Calu-3 cells. MDCK cells were infected with **(A)** Influenza A (H1N1pdm09), while Calu-3 cells were infected with **(B)** SARS-CoV-2; in both assays, cells were treated with concentrations ranging from 1.25 to 10 µM of the molecules**.** Cell viability was also evaluated in MDCK cells **(C)** and Calu-3 cells **(D)** after adding different concentrations of the compounds.
